# Differential transactivation of the upstream aggrecan enhancer regulated by PAX1/9 depends on SOX9-driven transactivation

**DOI:** 10.1038/s41598-019-40810-4

**Published:** 2019-03-14

**Authors:** Aki Takimoto, Chikara Kokubu, Hitomi Watanabe, Tetsushi Sakuma, Takashi Yamamoto, Gen Kondoh, Yuji Hiraki, Chisa Shukunami

**Affiliations:** 1 Laboratory of Cellular Differentiation, Institute for Frontier Life and Medical Sciences, Kyoto University, Kyoto, 606-8507 Japan; 2 Department of Genome Biology, Graduate School of Medicine, Osaka University, Suita, Osaka, 565-0871 Japan; 3 Laboratory of Integrative Biological Science, Institute for Frontier Life and Medical Sciences, Kyoto University, Kyoto, 606-8507 Japan; 4 Department of Mathematical and Life Sciences, Graduate School of Science, Hiroshima University, Higashi-Hiroshima, Hiroshima, 739-8526 Japan; 5 Department of Molecular Biology and Biochemistry, Division of Dental Sciences, Graduate School of Biomedical and Health Sciences, Hiroshima University, Hiroshima, 734-8553 Japan

## Abstract

A previously identified enhancer 10 kb upstream of the *Aggrecan* (*Acan*) gene (*UE*) can drive cartilage specific reporter expression *in vivo*. Here, we report that the paralogous transcription factors PAX1 and PAX9 differentially drive *UE*, depending on the presence or absence of SOX9-driven transactivation. In the developing vertebral column, PAX1/9 expression was inversely correlated with *Acan* expression. Moreover, PAX1/9 was co-expressed with SOX9/5/6 in the intervertebral mesenchyme and the inner annulus fibrosus (AF), and with SOX9 in the outer AF. Significant *Acan* upregulation was observed during chondrification of *Pax1*-silenced AF cells, while, *Acan* was significantly downregulated by persistent expression of *Pax1* in cartilage. Deletion of *UE* using CRISPR/Cas9 resulted in ~30% and ~40% reduction of *Acan* expression in cartilage and the AF, respectively. In the *UE*, PAX1/9 acts as weak transactivators through a PAX1/9-binding site partially overlapped with a SOX9-binding site. In the presence of SOX9, which otherwise drives robust *Acan* expression along with SOX5/6, PAX1/9 competes with SOX9 for occupancy of the binding site, resulting in reduced transactivation of *Acan*. Coimmunoprecipitation revealed the physical interaction of Pax1 with SOX9. Thus, transactivation of the *UE* is differentially regulated by concerted action of PAX1/9, SOX9, and SOX5/6 in a context-dependent manner.

## Introduction

Aggrecan (ACAN) is a chondroitin sulfate proteoglycan that is abundantly present in cartilage and the intervertebral discs (IVDs)^1^. Due to its hydrophilic properties, ACAN provides cartilage and the IVDs with a high osmotic swelling pressure to resist compressive loads. Especially in the vertebral column, the IVDs between vertebral bodies (VBs) with cartilaginous endplate at their top and bottom portions serve as cushions and allow various vertebral movements such as extension, flexion, and torsion^2^. The shock-absorbing gel of the nucleus pulposus (NP), containing abundant cartilaginous proteoglycans, is surrounded by the collagen-rich annulus fibrosus (AF). The inner AF (IAF) consists of several layers of fibrocartilage, while the outer AF (OAF) is a fibrous tissue containing highly organized fibers composed mainly of type I collagen, allowing it to resist tensile loads. Cartilaginous proteoglycans and type II collagen in the IAF decrease gradually closer to the OAF, while type I collagen content increases^3^.

*Acan* is induced and upregulated during chondrogenic differentiation and subsequent chondrocyte maturation. Traditional studies using transgenic reporter mice or zebrafish revealed that *Acan* transcription is redundantly regulated by several enhancers with or without the Sry-related HMG box (SOX) transcription factors^4,5^. Among them, the enhancer 10 kb upstream of the *Acan* gene (*UE*) can drive cartilage-specific reporter gene expression in transgenic mice and zebrafish, and its transactivation is mainly regulated by SOX9, SOX5, and SOX6^4,5^. The homeobox transcription factor SHOX interacts with SOX5 and SOX6 to activate the *UE*^6^.

The paralogous transcription factors PAX1 and PAX9, containing highly conserved paired box DNA-binding domains, are coexpressed with SOX9 in the sclerotome that gives rise to the axial skeleton^7^; however, their expression is later restricted to the AF of the IVD^8^. The complete lack of VBs and IVDs in *Pax1*/9 double-null mice suggests their indispensable roles in medioventral sclerotomal differentiation^9^. PAX1 acts as either a positive or negative regulator of *Acan* expression. PAX1 stimulates *Acan* expression via NKX3.2 to promote the early chondrogenesis of presomitic mesoderm explants^10,11^, while persistent expression of *Pax1* in chondrocytes downregulates *Acan* expression to result in dedifferentiation of chondrocytes^12^. These stage-specific actions of PAX1 in *Acan* expression and the restricted expression of PAX1/9 in the AF of IVDs suggest that PAX1/9 is also involved in *Acan* transcriptional regulation, but the molecular mechanism remains largely unknown.

Focusing on the *UE*, the upstream *Acan* enhancer, here, we demonstrate the fine-tuning of *Acan* expression in the *UE* by PAX1/9. Contribution of the *UE* to endogenous *Acan* transcription *in vivo* was evaluated by CRISPR/Cas9 mediated enhancer deletion. Through a PAX1/9-binding site that partially overlaps with a SOX9-binding site in the *UE*, PAX1/9 competes with SOX9 for occupancy of the binding site that results in a reduction in *Acan* transactivation, but in the absence of SOX9, PAX1/9 weakly transactivates the *UE*. These results suggest that PAX1/9 differentially drives the *Acan UE*, depending on the presence or absence of SOX9.

## Results

### Inverse correlation between PAX1/9 and ACAN expression in the IVDs

**Figure 1:**
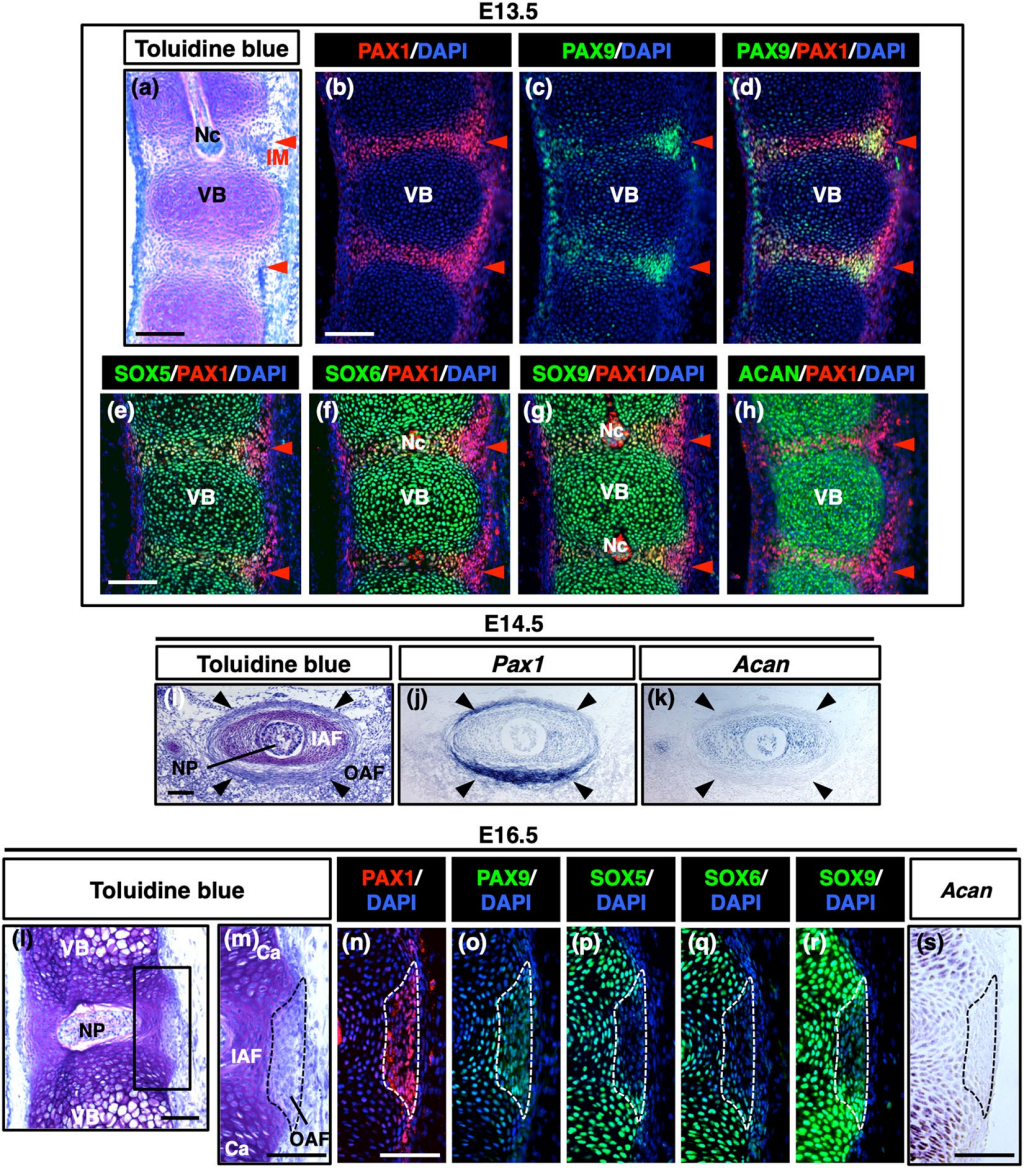
Differential expression of PAX1/9 and SOX9/5/6 in the intervertebral disc. (**a–h**) Sagittal sections of the thoracic vertebrae of a mouse embryo at E13.5 were processed for toluidine blue staining (**a**) and immunostaining for PAX1 (**b**,**d**,**e–h**), PAX9 (**c**,**d**), SOX5 (**e**), SOX6 (**f**), SOX9 (**g**), or ACAN (**h**). Red arrowheads in (**a–h**) indicate the IM that is positive for PAX1. (**i**–**k**) Cross sections of the thoracic IVD of a mouse embryo at E14.5 were processed for toluidine blue staining (**i**) or *in situ* hybridization for *Pax1* (**j**) and *Acan* (**k**). Black arrowheads in (**i**–**k**) indicate the OAF of the IVD. (**l**–**s**) Sagittal sections of the thoracic vertebrae of a mouse embryo at E16.5 were processed for toluidine blue staining (**l**,**m**); immunostaining for PAX1 (**n**), PAX9 (**o**), SOX5 (**p**), SOX6 (**q**), or SOX9 (**r**); and *in situ* hybridization for *Acan* (**s**). The boxed region in (**l**) is shown at a higher magnification in (**m**). Panels shown in (**n**–**s**) correspond to the area of **(m**). Dotted line in (**m**–**s**) encloses the OAF. Ventral is to the right in (**a**–**h**,**l**–**s**) or the bottom in (**i**–**k**). Ca, cartilage; IAF, inner annulus fibrosus; IM, intervertebral mesenchyme; Nc, notochord; NP, nucleus pulposus; OAF, outer annulus fibrosus; VB, vertebral body. Scale bars, 100 μm.

We analyzed the expression of PAX1, PAX9, SOX5, SOX6, SOX9, and ACAN in the developing mouse vertebral column from embryonic day (E) 13.5 to E16.5 by immunostaining (Fig. 1b–h,n–r) and *in situ* hybridization (Fig. 1j,k,s). At E13.5, cartilaginous proteoglycans that were detected by metachromatic staining with toluidine blue were abundantly present in the cartilaginous anlagen of VBs and were also present at a low level in the intervertebral mesenchyme (IM) (Fig. 1a). PAX1 was expressed in the IM and in the perichondrial tissues of VBs (Fig. 1b). PAX9 was co-localized with PAX1 in the IM and was also found in a portion of chondrocytes in the VBs (Fig. 1c,d). SOX9, SOX5, SOX6, and ACAN were distributed in the cartilaginous VBs (Fig. 1e–h). In the IM, PAX1 was co-localized with these molecules (Fig. 1e–h). At E14.5, the IVD was formed in association with the transition of the notochord into the NP. In the cross-sections of an IVD, the NP was surrounded by the AF, which is comprised of the IAF and the OAF (Fig. 1i). *Pax1* was strongly expressed in the OAF but weakly expressed in the IAF (Fig. 1j). In contrast, *Acan* was expressed in the IAF but only weakly expressed in the OAF (Fig. 1k). In the sagittal section of the vertebral column of an embryo at E16.5, significant accumulation of proteoglycans was detected in the cartilage and the IAF, while their accumulation in the OAF was nearly undetectable (Fig. 1l,m). At E16.5, PAX1 and PAX9 were present mainly in the OAF, but were also detected in a part of the IAF (Fig. 1n,o). Although SOX5 and SOX6 in the OAF were detectable only in the boundary to the IAF, SOX9 protein was weakly expressed throughout the entire OAF (Fig. 1p–r). *Acan* was expressed at a high level in the cartilage, and its expression gradually decreased from the IAF towards the OAF, which expressed PAX 1 and PAX9 (Fig. 1n,o,s). Thus, in the IVD, PAX1/9 expression is inversely correlated with ACAN expression and co-expressed with SOX9 in the OAF and with SOX9/5/6 in the IAF.

### Upregulation of *Acan* in Pax1-silenced AF cells during chondrification

**Figure 2:**
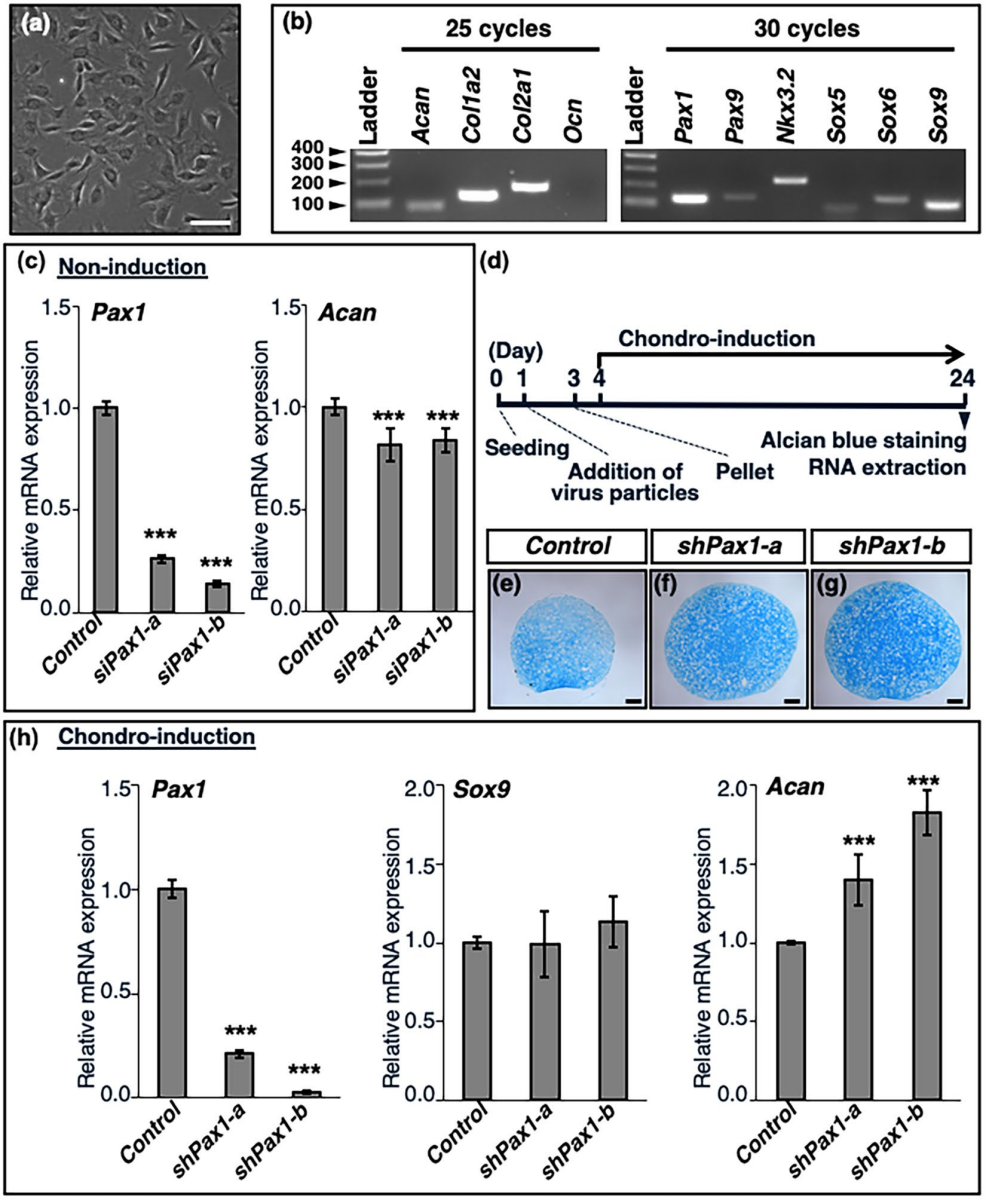
Enhanced chondrogenesis in pellets of AF cells by knockdown of *Pax1*. (**a**) AF cells isolated from 4-week-old Wistar rats were seeded at a density of 8 × 10^4^ cells/well in a 12-well plate. Morphology of AF cells cultured in DMEM containing 10% FBS at day 1 is shown. (**b**) Total RNA was extracted from AF cells maintained in a culture dish for 96 h and then processed for RT-PCR analysis of expression of ECM molecules (*Acan*, *Col1a2*, *Col2a1*, *Ocn*) and transcription factors (*Pax1*, *Pax9*, *Nkx3*.*2*, *Sox5*, *Sox6*, *Sox9*). Full-length gels are presented in Supplementary Fig. 7. (**c**) AF cells were seeded at a density of 4 × 10^4^ cells/well in a 24-well plate. At 24 h after inoculation, the cells were transfected with non-targeting siRNA (control) or *Pax1* siRNAs (*siPax1-a* or *siPax1-b*) by lipofection. Total RNA was extracted from the cells at 48 h after lipofection, and the expression levels of *Pax1* or *Acan* were examined by qRT-PCR. (**d**) Experimental schedule of chondro-induction in pelleted cultures of AF cells. AF cells were seeded at a density of 1 × 10^5^ cells/well in 6-well plates and then infected with shRNA lentiviruses on day 1. After trypsinization on day 3, cell pellets were prepared from 1.5 × 10^5^ cells/pellet and cultured in chondrogenic differentiation medium for another 21 days. (**e**–**g**) Sections of cell pellets infected with shRNA lentiviruses were stained with alcian blue. Increased accumulation of alcian blue-stained extracellular matrix was observed in the pellets of AF cells infected with *shPax1-a* or *shPax1-b* lentivirus (**f**,**g**) compared to that in cells infected with control lentivirus (**e**). (**h**) Total RNA was extracted from pellets on day 24. Relative expression levels of *Pax1*, *Sox9*, and *Acan* were examined by qRT-PCR analysis. qRT-PCR data (**c**,**h**) represent the average of three independent experiments. The relative expression of each gene is normalized to that in the control and reported as mean ± s.d. ***P < 0.001 versus control. Scale bars in (**a**) and (**e**–**g**), 100 μm.

In the IVD, an inverse correlation between *Pax1/9* and *Acan* expression was observed (Fig. 1h,j,k,n,s). Despite the abundant expression of SOX9 (Fig. 1g,r), the level of *Acan* in the OAF expressing high levels of PAX1/9 (Fig. 1d,n,o) was very low (Fig. 1k,s). We isolated AF cells from the IVDs of rat tails (Fig. 2a) and checked the expression of the genes encoding extracellular matrix proteins (*Acan*, *Col1a2*, and *Col2a1*) and transcription factors (*Pax1*, *Pax9*, *Nkx3*.*2*, *Sox5*, *Sox6*, and *Sox9*) (Fig. 2b), all of which have been reported to be expressed in the AF^2,8,13^. *Osteocalcin* (*Ocn*), used as a negative control, was confirmed to be undetectable (Fig. 2b). Since the expression level of *Pax1* was much higher than that of *Pax9*, we silenced *Pax1* in AF cells under non-inductive and chondro-inductive conditions. In AF cells, the level of *Pax1* was decreased to 26% or 14% of that in the control by *siPax1-a* or *siPax1-b*, respectively (Fig. 2c). Upon knockdown of *Pax1* by lipofection of the *siPax1* oligonucleotides, the mRNA levels of *Acan* were slightly but significantly decreased compared with those in the control (Fig. 2c). To investigate how *Pax1* affects the expression of *Acan* and proteoglycan accumulation during chondrogenic differentiation, AF cells infected with shRNA lentivirus were pelleted and maintained in chondrogenic differentiation medium containing l-ascorbic acid, dexamethasone, and recombinant human TGF-β1 (Fig. 2d). In the AF cells cultured under chondro-inductive conditions, the expression level of *Pax1* decreased by 21%, whereas that of *Sox5*, *Sox6*, or *Sox9* increased more than five-fold compared to those in the AF cells cultured under non-inductive conditions (Supplementary Fig. 1). Infection with *shPax1-a* or *shPax1-b* lentivirus resulted in increased accumulation of proteoglycans in the AF pellets as compared to levels in the control (Fig. 2e–g). The level of *Pax1* was decreased to 21% or 3% of that in the control by infection with *shPax1-a* or *shkPax1-b* lentivirus, respectively (Fig. 2h). Although no significant changes were detected in the level of *Sox9*, the level of *Acan* was significantly increased to 140% or 187% of that in the control by infection with *shPax1-a* or *shPax1-b* lentivirus, respectively (Fig. 2h). No significant increase in endogenous *Pax9* expression was detected in the pellets of AF cells infected with *shPax1-a* or *shPax1-b* lentivirus (Supplementary Fig. 2), suggesting that a compensatory increase in endogenous *Pax9* does not occur upon knock-down of *Pax1* in AF cells. Taken together, these results indicate that PAX1 stimulates *Acan* expression in AF cells under non-inductive conditions but inhibits its expression under chondro-inductive conditions.

### Downregulation of *Acan* in *Col2a1-Pax1* transgenic (*Tg*) mouse embryos

**Figure 3:**
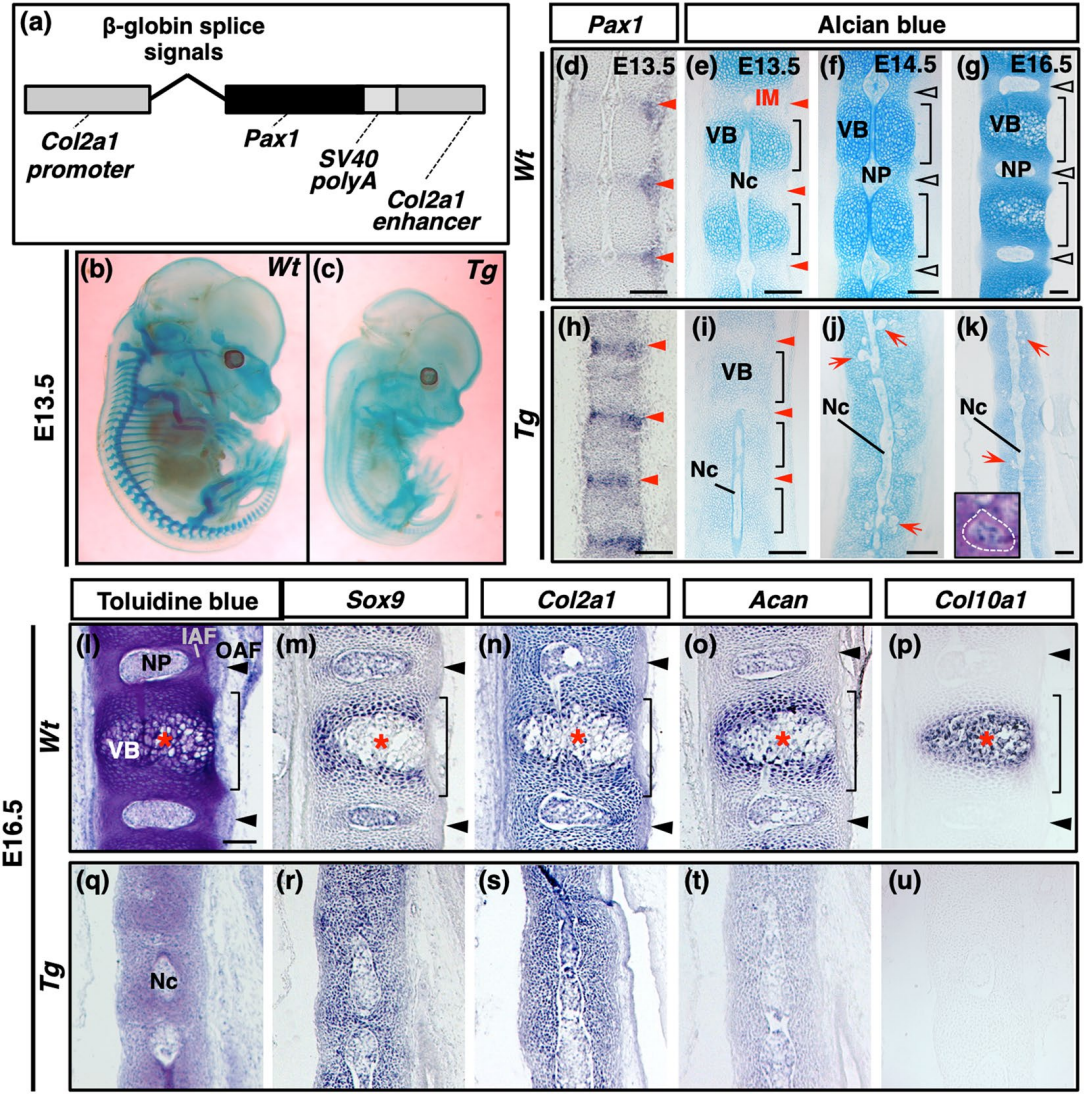
Impaired vertebral column formation in transgenic mouse embryos overexpressing *Pax1* in the *Col2a1* expressing region. (**a**) A schematic illustration of the transgene for generation of transgenic mice overexpressing *Pax1* under the control of *Col2a1* enhancer/promoter (*Col2a1-Pax1* Tg). (**b**,**c**) Lateral views of E13.5 *Col2a1-Pax1* Tg founder embryos stained with alcian blue are shown. Cartilaginous vertebrae, ribs, and limbs are stained with alcian blue in *Wt* (**b**), whereas accumulation of alcian blue-stainable matrix in those cartilaginous elements is significantly reduced in *Tg* (**c**). (**d**–**u**) Sagittal sections of thoracic vertebrae of *Wt* (**d–g**,**l**–**p**) or *Tg* (**h**–**k**,**q**–**u**) embryos at E13.5 (**d**,**e**,**h**,**i**), E14.5 (**f**,**j**), and E16.5 (**g**,**k**–**u**) are shown. The sections were processed for *in situ* hybridization of *Pax1* (**d**,**h**), *Sox9* (m,r), *Col2a1* (**n**,**s**), *Acan* (**o**,**t**) or *Col10a1* (**p**,**u**) and for alcian blue staining (**e-g**,**i**–**k**) or toluidine blue staining (**l**,**q**). Brackets in (**e–g**,**i**) and red arrowheads in (**d**,**e**,**h**,**i**) indicate the VB and the IM, respectively. Hollowed arrowheads in (**f**,**g**) and black arrowheads in (**l**–**p**) indicate the IVD and the OAF, respectively. Arrows in (**j**,**k**) indicate remnants of the notochord. The inset in (**k**) shows a magnified image of a toluidine blue stained notochordal remnant that is enclosed with a dotted line. Asterisks in (**l**–**p**) indicate the zone of hypertrophic chondrocytes. Ventral is to the right. IAF, inner annulus fibrosus; IM, intervertebral mesenchyme; Nc, notochord; NP, nucleus pulposus; OAF, outer annulus fibrosus; VB, vertebral body. Scale bars, 100 μm.

We previously reported that overexpression of *Pax1* resulted in downregulation of cartilage marker genes including *Acan* in the forelimbs of chicken embryos and cultured chondrocytes^12^. To examine the effects of persistent expression of *Pax1* in cartilage, we analyzed *Col2a1-Pax1 Tg* mouse embryos that overexpress *Pax1* under the control of *Col2a1* enhancer/promoter (Fig. 3a). Whole mount alcian blue staining revealed that *Col2a1-Pax1 Tg* mouse embryos were smaller and their cartilaginous tissues were weakly stained with alcian blue as compared to wildtype (*Wt*) littermates (Fig. 3b,c and Supplementary Fig. 3a–d). At E16.5, mineralized areas stained with alizarin red were detectable both in the membranous and the endochondral bones of wild type embryos, whereas the endochondral bones were not mineralized in *Col2a1-Pax1 Tg* embryos (Supplementary Fig. 3e–h). In the sagittal sections of the vertebral column of a *wt* embryo at E13.5, the notochord was observed as a rod-like structure running in the developing vertebral column in which the metameric pattern of VB and IM was established (Fig. 3d,e). From E14.5 onwards, the nucleus pulposus derived from the notochord was formed in the middle region of each IVD in *wt* embryos (Fig. 3f,g). In a *Col2a1-Pax1 Tg* embryo, *Pax1* was expressed in the VBs as well as the IM, which resulted in significant reduction of proteoglycan accumulation in the cartilaginous molds (Fig. 3h–k). Although the metameric pattern in the vertebral column of a *Col2a1-Pax1 Tg* embryo was observed by E13.5, such pattern was abolished with failure of IVD formation from E14.5 onwards (Fig. 3i–k). The vertebral column was ectopically bent at E14.5, and the notochord remained as a rod-like structure without forming the nucleus pulposus at E16.5 in *Col2a1-Pax1 Tg* embryos (Fig. 3i–k and Supplementary Fig. 3c). At E14.5 and E16.5, persistent notochordal remnants were found in the vertebral column of *Col2a1-Pax1 Tg* embryos (arrows in Fig. 3j,k). In the sagittal sections of the vertebral column of wild type embryos at E16.5, *Sox9*, *Col2a1*, and *Acan* were expressed both in the IVDs and the VBs, but these transcripts were downregulated in the hypertrophic chondrocytes expressing *Col10a1* (Fig. 3l–p). In a *Col2a1-Pax1 Tg* embryo, arrested chondrocyte maturation was manifested by lack of *Col10a1* expression as well as uniform expression of *Sox9* and *Col2a1* (Fig. 3q–u). Although the expression of *Sox9* and *Col2a1* in the cartilage was detected at a high level in a *Col2a1-Pax1 Tg* embryos, *Acan* expression was detected at a lower level than that in the cartilage of wild type embryos (Fig. 3r–t).

Thus, persistent expression of *Pax1* in the developing cartilage inhibits *Acan* expression and proteoglycan accumulation, suggesting that downregulation of *Pax1* is necessary for the proper progression of chondrocyte maturation during vertebral column development.

### Decreased endogenous *Acan* expression in the AF and the costal cartilage by *in vivo* deletion of the genome region containing the upstream *Acan* enhancer

**Figure 4:**
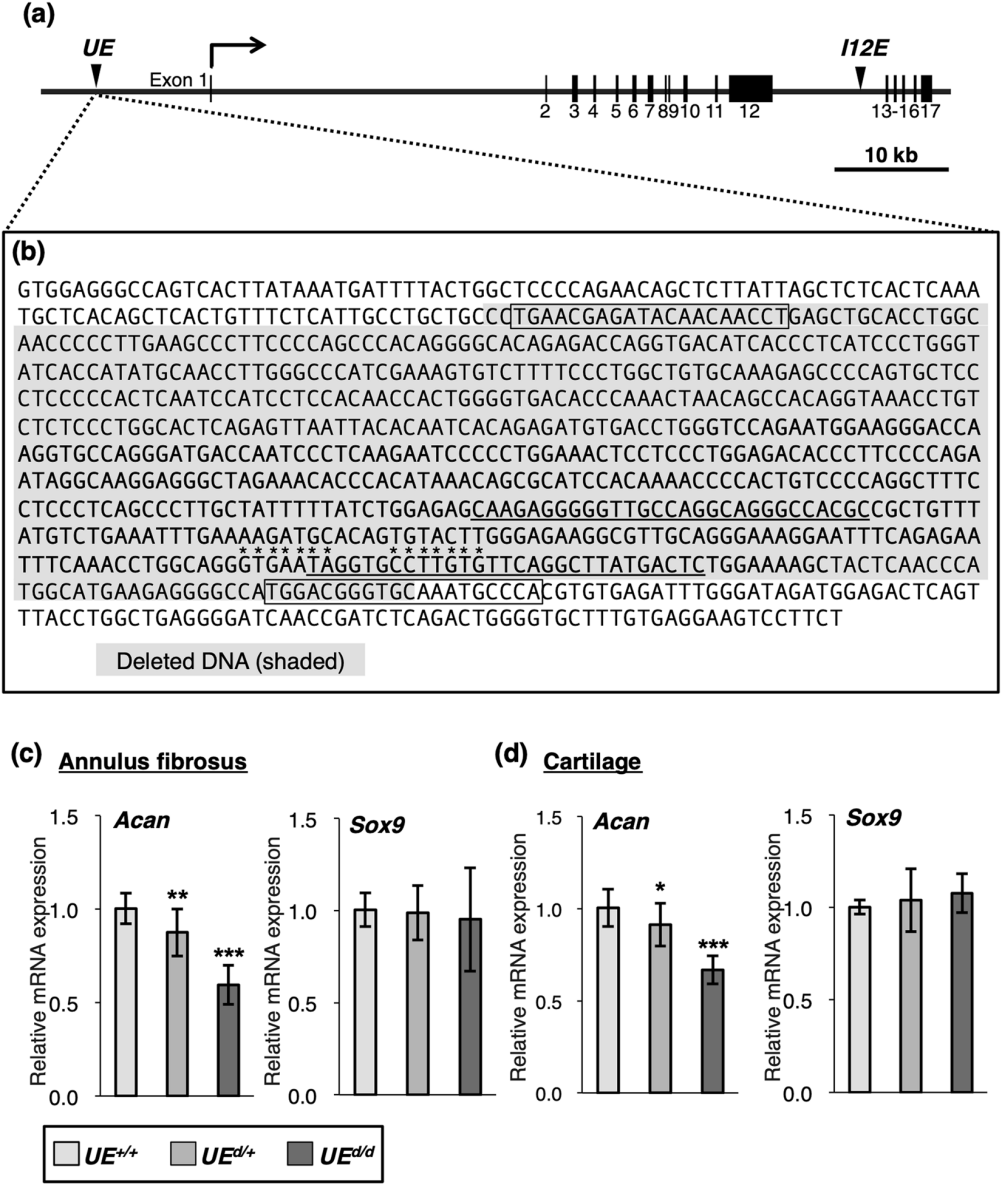
*In vivo* deletion of the *Acan* upstream enhancer. (**a**) Genomic structure of the mouse *Acan* gene, consisting of 17 exons (black boxes). Black arrowheads indicate the positions of the upstream enhancer (*UE*), located approximately 10 kb upstream of the transcription start site, and of the *I12* enhancer (*I12E*) located in intron 12. (**b**) The mouse DNA sequence surrounding the *UE*. The sequence of the *UE* is shown in bold letters. Two potential binding sites for PAX2/5/8, predicted by MatInspector software (Genomatix), are underlined. The shaded letters indicate the region of a deletion mutation produced by the CRISPR/Cas system. The boxed letters indicate the sgRNA target sequence. The bases comprising the SOX9-binding sites are indicated with asterisks. (**c**,**d**) Total RNA was extracted from the AF (**c**) and the costal cartilage (**d**) of *UE*^+*/*+^, *UE*^*d/*+^, or *UE*^*d/d*^ male mice at 3 weeks after birth. Relative expression levels of *Acan* and *Sox9* were examined by qRT-PCR analysis. qRT-PCR data represent the average of 4 mice in each group. The relative expression of each gene is normalized to that of *UE*^+*/*+^ and reported as mean ± s.d. *P < 0.05 versus *UE*^+*/*+^, **P < 0.01 versus *UE*^+*/*+^, ***P < 0.001 versus *UE*^+*/*+^.

Han and Lefebvre^5^ have previously identified the upstream *Acan* enhancer (*UE*) harboring SOX9- and SOX5/6-binding sites as a major *cis*-regulatory element for *Acan* expression (Fig. 4a). We used MatInspector software (Genomatix) to identify two potential binding sites for PAX2/5/8 (Fig. 4b). We then generated mutant mice lacking the SOX9-binding sites of the enhancer using clustered regularly interspaced short palindromic repeat (CRISPR)/CRISPR-associated protein 9 (Cas9) to evaluate its contribution to endogenous *Acan* expression (Fig. 4a,b). We established a mouse line with a deletion of a 695-bp region containing 359 bp of the *UE* (Fig. 4b). The expression level of *Acan* in the AF of the IVD was significantly reduced by 12% in heterozygous *UE*^*d/*+^ mice and by 41% in homozygous *UE*^*d/d*^ mice (Fig. 4c) compared to that in wildtype *UE*^+*/*+^ mice. In the costal cartilage, the *Acan* expression level was decreased by 9% in heterozygous *UE*^*d/*+^ mice and by 33% in homozygous *UE*^*d/d*^ mice compared to that in wildtype *UE*^+*/*+^ mice (Fig. 4d). The *in vivo* deletion of the *UE*-containing region did not significantly affect Sox9 expression in either tissue (Fig. 4c,d). These results suggest that the *UE* is a major enhancer that regulates *Acan* expression in the AF of the IVD and the costal cartilage.

### Transactivation of the upstream enhancer and the intragenic region regulated by PAX1/9, SOX9, and SOX5/6

**Figure 5:**
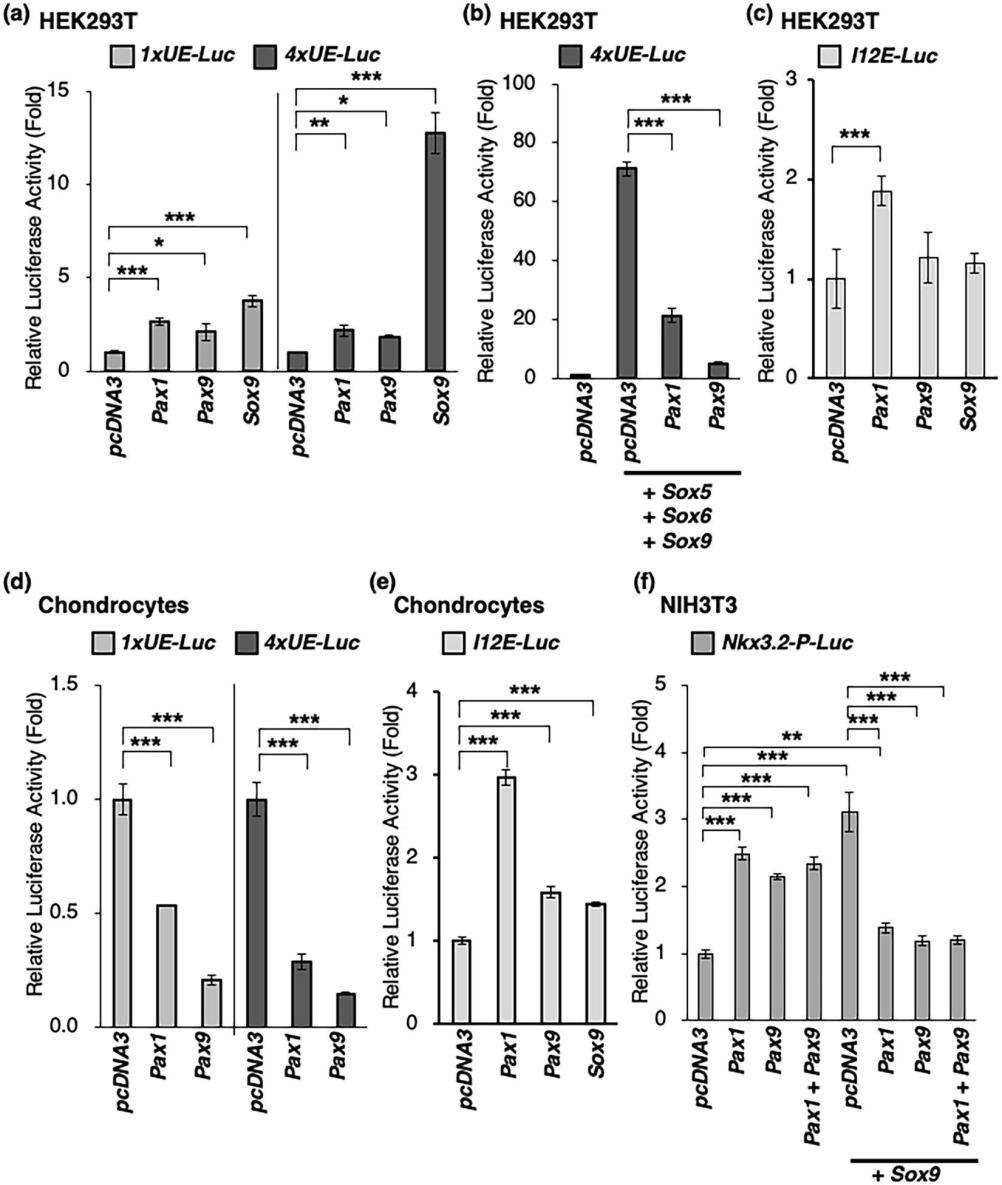
Transactivation of *Acan* enhancers by PAX1/PAX9 and SOX9/SOX5/SOX6. (**a–f**) Dual luciferase assays were performed with HEK293T cells (**a–c**), chondrocytes (**d**,**e**), or NIH3T3 cells (**f**). Cells were co-transfected with reporter plasmids (*pGL4*.*74 [hRluc/TK]* and *pGL4*.*23[luc2/minP]* or *pGL3-Basic* vectors) and expression plasmids (*pcDNA3* vectors). The *pGL4*.*23[luc2/minP]* and *pGL3-Basic* vectors used for the assays were *pGL4*.*23[luc2/minP]-1xUE-Luc* (*1xUE-Luc*), *pGL4*.*23[luc2/minP]-4xUE-Luc* (*4xUE-Luc*), or *pGL4*.*23[luc2/minP]-I12E-Luc* (*I12E-Luc*), and *pGL3-Basic-Nkx3*.*2-P-Luc* (*Nkx3*.*2-P-Luc*). The expression plasmids used were *pcDNA3* empty vector (*pcDNA3*), *pcDNA3-hSox5* (*Sox5*), *pcDNA3-hSox6* (*Sox6*), *pcDNA3-mSox9* (*Sox9*), *pcDNA3-FLAG-Pax1* (*Pax1*), or *pcDNA3-FLAG-Pax9* (*Pax9*). For the co-transfection of *pcDNA3* vectors in (**b**), 10 ng each of *Sox5*, *Sox6*, and *Sox9*, and 20 ng of *pcDNA3*, *Pax1*, or *Pax9* were used. For the co-transfection of *pcDNA3* vectors in (**f**), 25 ng each of *Pax1* and *Pax9* (the fourth bar from the left), or 25 ng of *Sox9* and 25 ng of *pcDNA3*, *Pax1*, or *Pax9* (from the fifth to the seventh bars from the left), or 12.5 ng each of *Pax1* and *Pax9* (the right end bar) were used. The firefly and *Renilla* luciferase activities were measured 24 h after transfection. Values were normalized using a *pGL4*.*74[hRluc/TK]* vector and are presented as fold induction relative to *pcDNA3* co-transfected with reporter plasmids. Graphs show a representative experiment out of at least three. Each bar represents the average of three independent transfections (means ± s.d.). *P < 0.05 versus *pcDNA3*, **P < 0.01 versus *pcDNA3*, ***P < 0.001 versus *pcDNA3* (**a**,**c**,**d**,**e**,**f**), *pcDNA3* + *Sox5* + *Sox6* + *Sox9* (**b**), or *pcDNA3* + *Sox9* (**f**).

To test whether *Acan* is a direct target gene of PAX1 or PAX9, we performed dual luciferase reporter assays using human embryonic kidney (HEK) 293T cells, primary chondrocytes, and NIH3T3 cells. One copy or four tandem repeats of 359 bp of the mouse *UE* (*1xUE-Luc* and *4xUE-Luc*) was inserted into the *pGL4*.*23[luc2/minP]* vector, containing a minimal promoter and a luciferase (*luc*) reporter gene. Co-expression of *Pax1*, *Pax9*, or *Sox9* with *1xUE-Luc* in HEK293T cells resulted in a 2.7-, 2.1-, or 3.8-fold increase in luc activity compared to that with the *pcDNA3* empty vector (Fig. 5a). The luc activity of *4xUE-Luc* was significantly increased by *Sox9* (12.8-fold), while luc activity was increased 2.2- or 1.9-fold when cells were co-expressed with *Pax1* or *Pax9* (Fig. 5a). In the presence of *Sox9* and *Sox5/6*, the luc activity of *4xUE-Luc* in HEK293T cells increased to 71-fold, but it decreased to 30% or 7% upon co-expression with *Pax1* or *Pax9*, respectively (Fig. 5b). We also identified a genomic region containing a potential PAX2-binding site in intron 12 (*I12E*) of the mouse *Acan* gene (Supplementary Fig. 4a,b). Gel shift assays revealed that PAX1 and PAX9, but not SOX9, bound to the predicted PAX2-binding site in *I12E* (Supplementary Fig. 4c–e). When co-expressed with *Pax1*, the luc activity of *I12E* (*I12E-Luc*) was significantly increased in HEK293T cells (Fig. 5c). In chondrocytes expressing *Sox9* and *Sox5/6* at high levels, the luc activities of *1xUE-Luc* and *4xUE-Luc* increased to 19- and 52-fold that of the *pGL4*.*23[luc2/minP]* empty vector, respectively (Supplementary Fig. 5). As expected, the luc activity of *1xUE-Luc* co-expressed with *Pax1* and *Pax9* decreased to 54% and 21%, respectively. Similarly, the luc activity of *4xUE-Luc* co-expressed with *Pax1* and *Pax9* decreased to 29% and 15%, respectively (Fig. 5d). In contrast, when the *I12E* fragment with the PAX1/9-binding site not overlapped with a SOX9-binding site was tested, PAX1 and PAX9 acted as transactivators in chondrocytes (Fig. 5e). Interestingly, in the previously identified *Nkx3*.*2* promoter region (*Nkx3*.*2-P*), which contains a PAX1/PAX9-binding site that partially overlaps with a SOX9-binding site^10,14^, SOX9-induced transactivation was significantly decreased by co-expression with PAX1 or PAX9 (Fig. 5f). Taken together, these results suggest that PAX1 and PAX9 act as weak transactivators in the transcriptional regulation of *Acan* and *Nkx3*.*2*. However, SOX9- or SOX9/5/6-induced transactivation of *Acan* and *Nkx3*.*2* expression is attenuated by PAX1 and PAX9, as the PAX1/9-binding sites partially overlap with the SOX9-binding sites.

### Competitive binding of PAX1/9 and SOX9 in the UE

**Figure 6:**
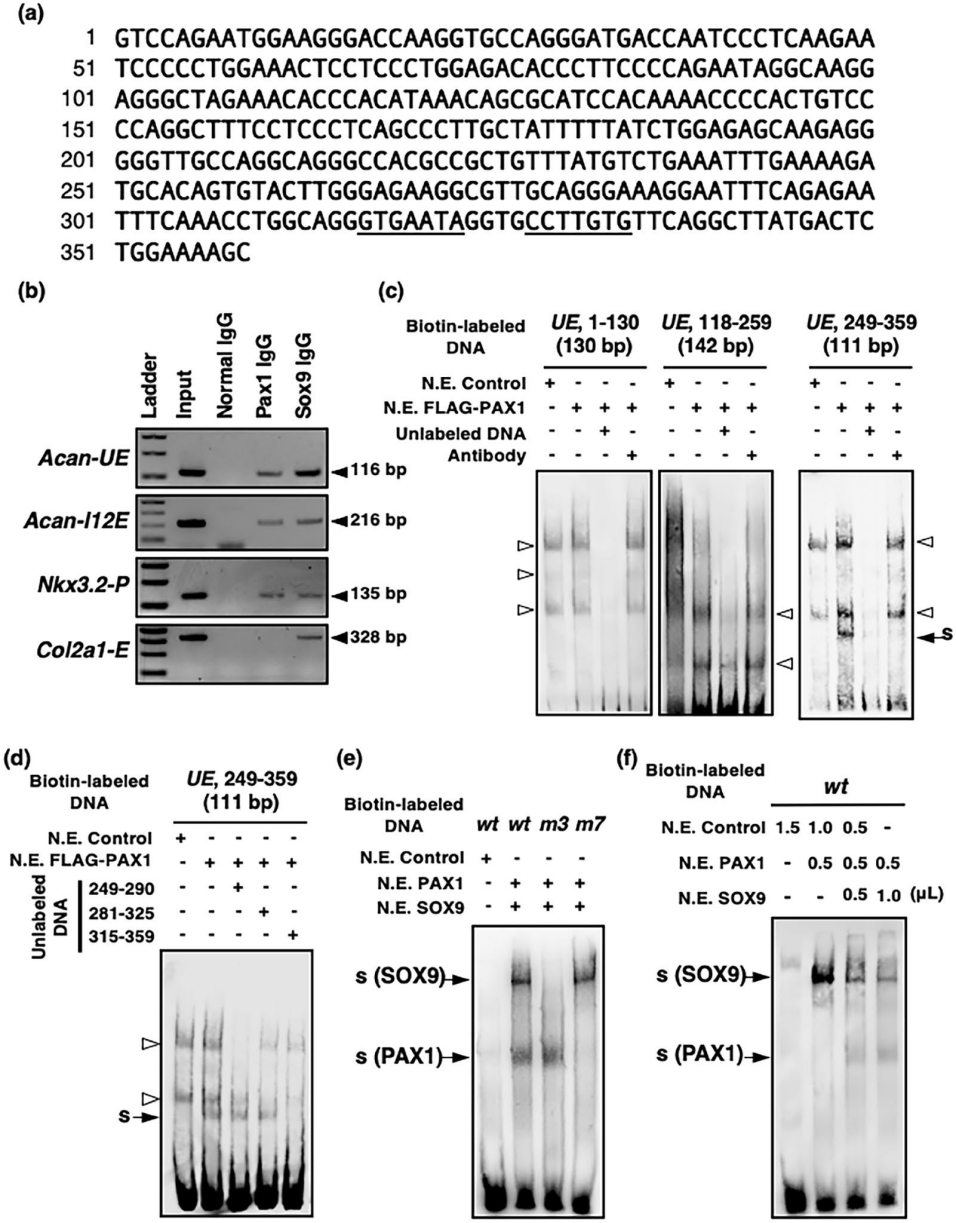
Binding of PAX1 to the 3′ region of the *UE*. (**a**) A 359-bp DNA sequence of the mouse *UE*. The SOX9-binding sites are underlined. (**b**) ChIP assays on extracts from AF cells. After immunoprecipitation of the cross-linked extracts with anti-PAX1 antibody, anti-SOX9 antibody, or normal IgG antibody, the DNA was subjected to PCR with primers that amplify a 116-bp fragment of the *UE*, 216-bp fragment of the *I12E*, 135-bp fragment of *Nkx3*.*2-P*, or 328-bp fragment of the *Col2a1-E*. Full-length gels are presented in Supplementary Fig. 7. (**c**,**d**) Gel shift assays were performed using 20 fmol of biotin-labelled dsDNA probes, 20 pmol of unlabeled dsDNA fragments, and nuclear extracts (N.E.) of HEK293T cells transfected with *pcDNA3* empty vector (N.E. Control) or *pcDNA3-FLAG-Pax1* (N.E. FLAG-PAX1). The biotin-labelled and unlabeled DNA fragments correspond to positions 1–130, 118–259, and 249–359 of the *UE*. A shifted band was caused by interactions between FLAG-PAX1 and the biotin-labelled 249–359 of *UE*. The shifted band in (**c**) disappeared in the presence of the unlabeled 249–359 oligonucleotide or anti-FLAG antibody (Antibody). The shifted band in (**d**) disappeared in the presence of the unlabeled 315–359 oligonucleotide but remained detectable in the presence of the unlabeled 249–290 or 281–325 oligonucleotide. (**e**,**f**) Gel shift assays were performed using biotin-labelled oligonucleotides shown in Supplementary Table 1 and nuclear extracts (N.E.) of HEK293T cells transfected with *pCAG* empty vector (N.E Control), *pCAG-Sox9* (N.E. SOX9), or *pCAG-Pax1* (N.E. PAX1). For the assays in (**e**,**f**), 100 and 5 fmol of the biotin-labelled oligonucleotides were used, respectively. A total of 1.5 μL of N.E. was used for each binding reaction in (**f**). A shifted band is shown with an arrow. Non-specific bindings are shown with open arrowheads. s, shifted band.

A potential binding site for PAX2/5/8 was found near the previously identified SOX9-binding sites located from 317 to 334 in the *UE* (Figs 4b and 6a). Chromatin immunoprecipitation (ChIP) assays revealed that PAX1 directly binds to the *UE* and *I12E* of *Acan* (*Acan-UE* and *Acan-I12E*) and the promoter region of *Nkx3*.*2* (*Nkx3*.*2-P*), as shown in Fig. 6b. We then performed gel shift assays to determine whether PAX1 and PAX9 directly bind to the predicted binding site that partially overlaps with the SOX9-binding sites found in the 359-bp *UE*. We tested three DNA fragments corresponding to the sequences at 1–130, 118–259, and 249–359 and identified the specific binding of PAX1-containing nuclear extracts to the 111-bp of the 3′-fragment of the *UE* (Fig. 6c). This specific binding competed with the non-labelled 45-bp oligonucleotide encoding the sequence at 315–359, but it was not affected by the non-labelled 249–290 or 281–325 oligonucleotides (Fig. 6d). We also confirmed the specific binding of PAX1 and PAX9 to the predicted binding site found in intron 12 (Supplementary Fig. 4c,d). In the *UE*, PAX1 bound the biotin-labelled 55-bp oligonucleotide (*wt*) corresponding to the sequence at 301–355 in the *UE*, which was consistent with the *in-silico* prediction of the potential PAX2/5/8-binding site at 322–350 (Fig. 4b, Supplementary Fig. 6a). Gel shift assays with mutated oligonucleotides revealed that PAX1 bound the 334–351 sequence in the *UE*, partially overlapping with the SOX9-binding site (Supplementary Fig. 6a). We also confirmed the binding of PAX9 to the *UE* by detection of the specific binding of PAX9 protein to the *wt* oligonucleotide and *m3* oligonucleotide (containing a mutated SOX9-binding site), but not to the *m7* oligonucleotide (containing a mutated PAX1-binding site), as was observed for PAX1 (Supplementary Fig. 6b).

The binding site for PAX1 partially overlapped with that for SOX9, raising the possibility that PAX1 interferes with the binding of SOX9 in the *UE*. To test this possibility, we performed gel shift assays using nuclear extracts from HEK293T cells expressing PAX1 or SOX9. In the presence of both PAX1 and SOX9 with biotin-labelled *wt* oligonucleotide, containing the two SOX9-binding sites and a PAX1/9-binding site, two specific bands were detected (lane 2 in Fig. 6e). When biotin-labelled *m3* oligonucleotide, containing a mutated SOX9-binding site, or *m7* oligonucleotide, containing a mutated PAX1/9-binding site, was used, specific shift bands for PAX1 and SOX9 were detected as PAX1/*m3* and SOX9/*m7* complexes, respectively (lanes 3 and 4 in Fig. 6e). A shift band for SOX9 (lane 2 in Fig. 6f) was detected in the presence of the *wt* oligonucleotide. When incubated with both SOX9 and PAX1, the intensity of the shift band for SOX9 was reduced, and instead the shift band for PAX1 was detected (lanes 3 and 4 in Fig. 6f). These data suggest that PAX1 and SOX9 compete for the partially overlapping binding sites in the *UE*.

**Figure 7:**
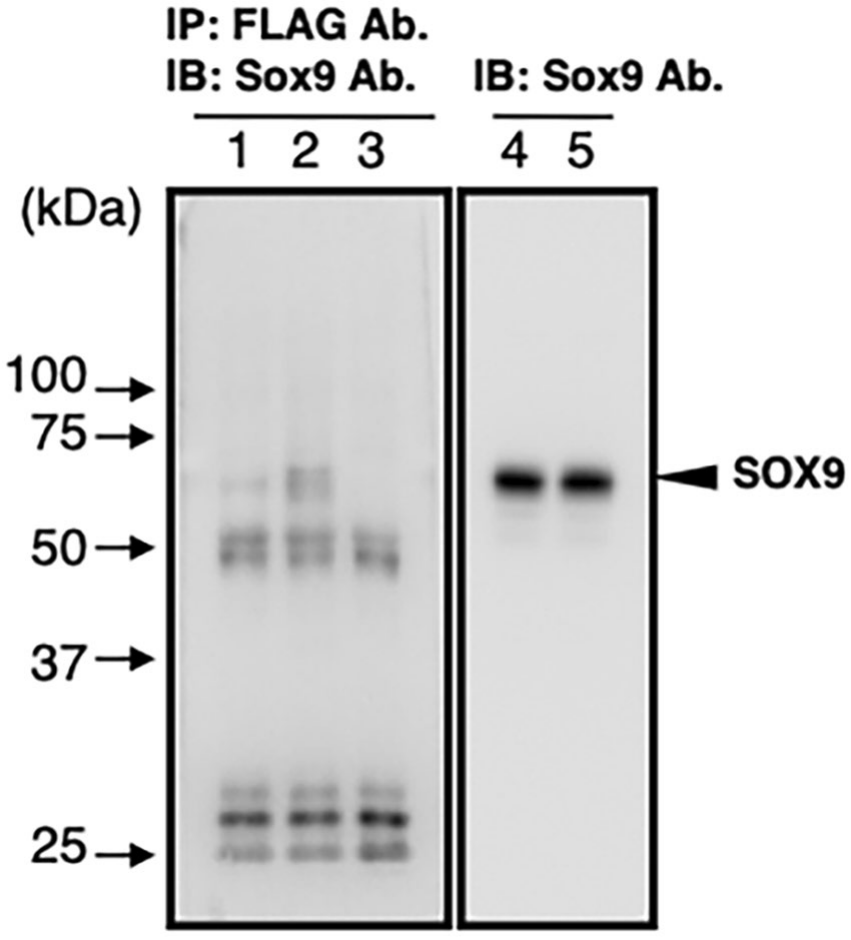
Physical interaction between FLAG-PAX1 and SOX9. Coimmunoprecipitation assays were performed with (lanes 1, 2) or without (lane 3) nuclear extracts. The nuclear extracts prepared from HEK293T cells co-transfected with *pcDNA3-Sox9* and *pcDNA3-FLAG-Runx2* or *pcDNA3-FLAG-Pax1* were immunoprecipitated with the anti-FLAG antibody. The immunoprecipitates (lanes 1–3) and the nuclear extracts (lanes 4, 5) were subjected to immunoblotting with the anti-Sox9 antibody. The band representing the SOX9 protein is indicated by the arrowhead on the right. Lane 1, immunoprecipitates of FLAG-RUNX2 and SOX9; lane 2, immunoprecipitates of FLAG-PAX1 and SOX9; lane 3, immunoprecipitates of the buffer for nuclear extraction; lane 4, nuclear extracts of the cells expressing FLAG-RUNX2 and SOX9; lane 5, nuclear extracts of the cells expressing FLAG-PAX1 and SOX9.

A previous study demonstrated that SOX9 directly interacted with RUNX2 to repress its activity^15^. To explore the possibility of a physical interaction between PAX1 and SOX9, we performed coimmunoprecipitation assays using the nuclear extracts of HEK293T cells transfected with *pcDNA3-Sox9* and *pcDNA3-FLAG-Pax1*. When the nuclear extracts were immunoprecipitated with anti-FLAG antibody, SOX9 was detected in the immunoprecipitates of the nuclear extracts containing FLAG-PAX1 and SOX9, and the nuclear extracts containing FLAG-RUNX2 and SOX9 (Fig. 7). These results suggest that the physical interactions between PAX1 and SOX9 interfere with each other to reduce their transcription activities. Thus, the extent of SOX9-driven transactivation of the *UE* is influenced by the presence or absence of PAX1.

## Discussion

**Figure 8:**
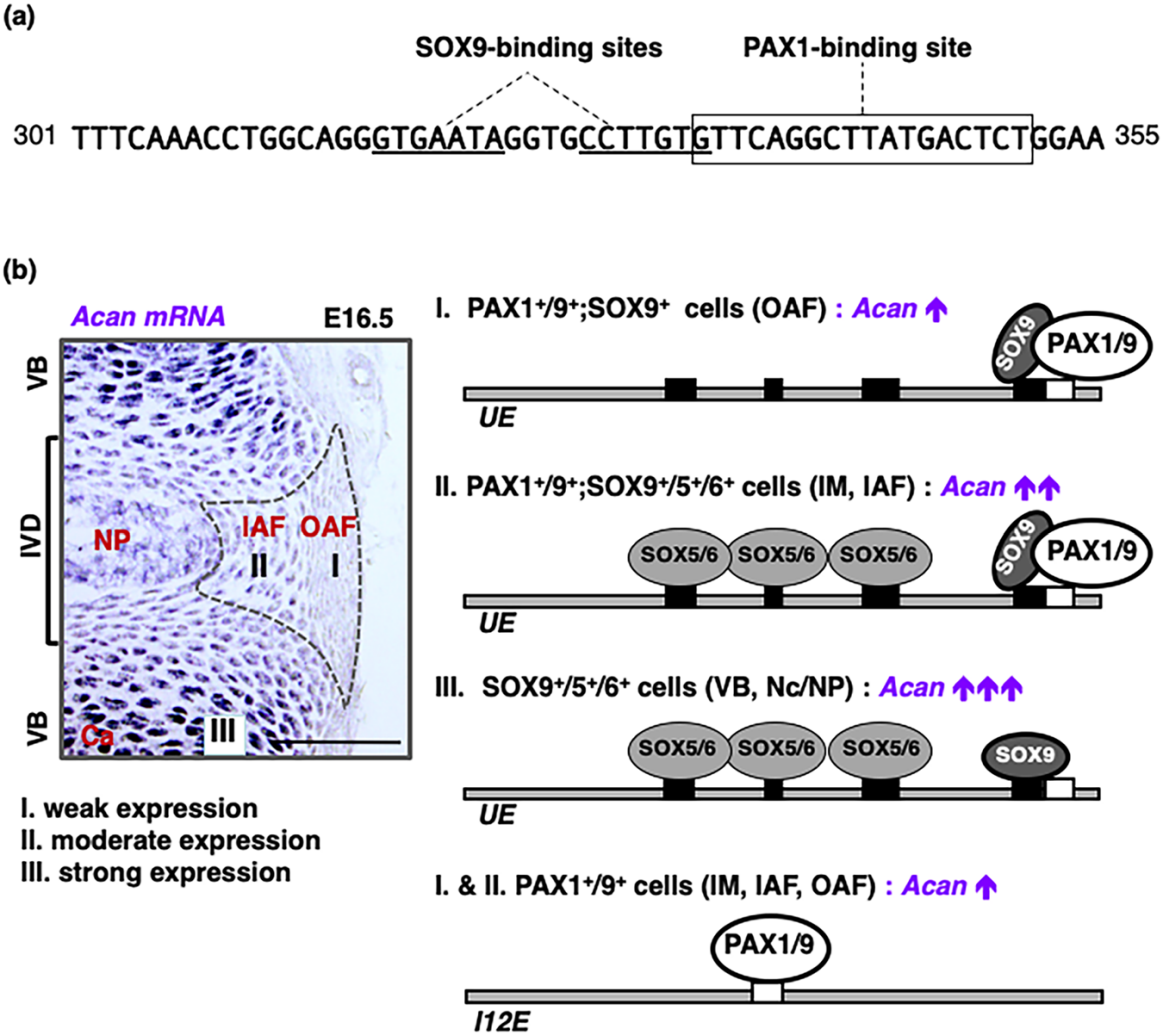
Differential *Acan* expression achieved by unique combinations of PAX1/PAX9, SOX9, and SOX5/SOX6. (**a**) The mouse *UE* sequence from 301 to 355 is shown. Underlined and boxed letters in the sequence are the SOX9-binding sites and the PAX1-binding site, respectively, as revealed by gel shift assays in Supplementary Fig. 6. (**b**) A proposed model for the transcriptional regulation of *Acan* mediated by the interaction of PAX1/9, SOX9, and SOX5/6 with the *Acan* enhancers is shown. During vertebral column development, *UE* weakly activates *Acan* transcription in the OAF (I), which expresses PAX1/9 and SOX9; moderately in the IAF (II), which expresses PAX1/9 and SOX9/5/6; and strongly in the cartilaginous VB (III), which expresses SOX9/5/6. In parallel, *I12E* activates *Acan* transcription weakly in the OAF and IAF, which express PAX1/9. Details are discussed in the text. Ca, cartilage; IAF, inner annulus fibrosus; IM, intervertebral mesenchyme; IVD, intervertebral disc, Nc, notochord; NP, nucleus pulposus; OAF, outer annulus fibrosus; VB, vertebral body. Scale bar, 100 μm.

In this study, our CRISPR/Cas9 mediated enhancer deletion *in vivo* demonstrated for the first time that the upstream *Acan* enhancer, *UE*, accounts for ~40% and ~30% of endogenous *Acan* expression in the AF and costal cartilage, respectively. In the developing vertebral column, we found an inverse correlation between PAX1/9 and *Acan* expression. PAX1/9 acted as weak transactivators of both the intragenic (intron 12) and the *UE*. However, in the presence of SOX9, which directs modest but robust transcriptional activation together with SOX5/6, PAX1 and SOX9 physically interacted with each other and competed for occupancy of their partially overlapping binding sites within the *UE* (Fig. 8a), thus resulting in reduced transactivation (Fig. 8b). These results suggest that unique combinations of PAX1/9, SOX9, and SOX5/6 contribute to the establishment of the graded expression and accumulation of ACAN.

During vertebral column development, the formation of IVDs is associated with the physical transition of the rod-like notochord into nuclei pulposi^16^. No residual notochordal tissue normally remains in the vertebral body. In the *Col2a1-Pax1 Tg* embryos, the notochord remained as a rod-like structure without forming the nucleus pulposus nor IVDs. This was similarly observed in the vertebral column of *Col2a1*^−/−^ mouse embryos^17^. It has been proposed that swelling pressure exerted by the cartilaginous vertebral bodies serves to push notochord cells into the intervertebral regions during the transition of the notochord into nuclei pulposi^4^. Our results suggest that persistent expression of *Pax1* in *Col2a1-Pax1 Tg* downregulates *Acan* expression to induce dedifferentiation of chondrocytes, reduced matrix synthesis and accumulation in cartilage, the notochord, and the notochord sheath, thus resulting in a failure of the normal transition of the notochord into nuclei pulposi.

The silencing of *Pax1* in AF cells isolated from the IVD of 4-week-old rats resulted in the slight downregulation of *Acan* expression. This is in accordance with the previous observation that the loss of *Pax1* causes a decrease in *Acan* expression^8^. However, significant accumulation of proteoglycan and upregulation of *Acan* gene expression were observed in *Pax1*-silenced AF cell pellets under chondro-inductive conditions, indicating that PAX1 acts as a negative regulator of *Acan* expression during chondrification. This is consistent with our previous and present findings that *Acan* expression is markedly downregulated in chondrocytes upon forced expression of *Pax1* in chicks^12^ and transgenic mice. Since there were no significant changes in *Sox9* mRNA levels, and no increase in those of *Pax9*, in the control vs. the *Pax1*-silenced AF pellets under chondrogenic conditions, reduced *Pax1* expression could ultimately lead to augmented transcriptional activity of SOX trio (SOX5, 6, 9) complexes, which drive robust *Acan* expression.

When two transcription factors compete for shared DNA binding sites, robust transactivation can be blocked by enhancer or promoter occupancy^18^. This mode of regulation has been proposed for several combinations of transcription factors, such as HMX1 and NKX2.5, PAX3 and MITF, and SP1 and SP3^19–21^. Such antagonistic regulation can be mediated not only by a combination of a transcriptional activator and repressor, but also by a combination of activators, such as SP1 and SP3^21^. We found that PAX1/9 competes with SOX9 for occupancy of the shared binding sites within the *UE* (Fig. 8a). In HEK293T cells, where neither SOX9 nor SOX5/6 is expressed, PAX1/9 and SOX9 transactivated the *Acan* upstream enhancer (*4xUE-Luc*) ~2-fold and ~12-fold, respectively. When co-expressed with SOX9 and SOX5/6, the luc activity of *4xUE-Luc* was ~70-fold greater than that of the control. Either PAX1 or PAX9 can attenuate this transactivation driven by SOX9 and SOX5/6 through decreased binding of SOX9 to the shared binding site. Consistent with these observations, luc activity of *1xUE-Luc* or *4xUE-Luc* decreased when PAX1 or PAX9 was expressed in chondrocytes which express SOX9 and SOX5/6 endogenously at high levels. Both PAX1 and PAX9 compete with SOX9 for occupancy of the partially overlapping binding sites, resulting in reduced transactivation of *Acan* in chondrocytes. This suggests that a unique molecular mechanism operates in non-hyaline cartilaginous tissues such as the AF, in which weak and strong transactivators cooperatively contribute to the fine-tuning of *Acan* expression by changing their relative expression ratios.

The *Acan UE* was identified as an evolutionarily conserved genomic region that drives cartilage-specific reporter gene expression in transgenic mice^5^. Later, Hu *et al*. identified multiple *Acan* enhancers, whose activities were analyzed in zebrafish^22^. In these studies, transgenic reporter assays using a single copy or tandem copies of the candidate enhancer regions were performed to evaluate the transactivation potentials and tissue specificities of these enhancers. Accumulating evidence has suggested that the pervasive presence of multiple enhancers with similar activities near the same gene confers phenotypic robustness against loss-of-function mutations in individual enhancers^23^. Deletion of an individual enhancer would cause no noticeable change, even though it drives reporter gene expression in transgenic animals^23^. However, our *in vivo* deletion analysis of the *Acan UE* demonstrated that ~40% of the endogenous *Acan* expression in the AF is driven by this enhancer. Considering that even subtle changes in *Acan* expression levels could cause degeneration of the IVD or osteoarthritis, the *Acan UE*, the activity of which is finely tuned by combinations of strong and weak transactivators, is considered to be important in maintaining vertebral column integrity in the postnatal IVD.

Our study revealed a novel PAX1/9-binding motif, 5′ GT**TCA**G**GC**TTA**TGA**CTCT 3′, which contains the core sequence of a PAX2-binding motif, 5′ **TCA**N**GC**N**TGA** 3′^24^. In the mouse *Acan UE*, the PAX1-binding site partially overlapped with one of the SOX9-binding sites. Similar PAX1/9-binding motifs that partially overlap with SOX9-binding sites are also present in the *Acan UE* in other mammals (data not shown). Interestingly, it has been reported that a PAX1/9-binding site partially overlaps with the SOX9-binding site in the mouse *Nkx3*.*2* promoter^14^. In *Pax1/9*-deficient mice, *Nkx3*.*2* expression is undetectable in the sclerotome^10^. During early chondrogenesis, PAX1/9 induces the expression of *Nkx3*.*2*, which stimulates *Acan* expression in explants of the chick presomitic mesoderm^10^. In contrast, persistent expression of *Pax1* and *Nkx3*.*2* in chick chondrocytes results in significant downregulation of *Acan*^12^. PAX1/9 physically interacts with the *Nkx3*.*2* promoter to transactivate it^10^. Transactivation of the *Nkx3*.*2* promoter by SOX9 is significantly suppressed by PAX1 or PAX9, suggesting a common mechanism in which SOX9-driven transactivation is modulated by PAX1 or PAX9 through competition for overlapping binding sites and physical interactions.

The OAF is a fibrous tissue that still expresses *Acan* at a low level. It is of interest that the OAF predominantly expresses not only *Pax1/Pax9*, but also *Scx*, which was also reported to be a transactivator of *Acan*^25^. In *Pax1/Pax9* double-null mice, IVD is completely missing^9^. Loss of *Scx* also causes defective formation of the OAF during development^26^. We previously reported that the conditional inactivation of *Sox9* in SCX^+^/SOX9^+^ cells caused defective formation of the IAF but expansion of the OAF^27^, suggesting that the SCX^+^/SOX9^+^ cell population contributes to the formation of the AF. IAF formation is delayed in *Sox5*^−/−^*/Sox6*^−/−^ mice, in which *Acan* expression is almost undetectable^4^. ACAN content increases from the PAX1^+^/SOX9^+^ OAF towards the PAX1^+^/SOX9^+^/SOX5^+^/SOX6^+^ IAF and the central SOX9^+^/SOX5^+^/SOX6^+^ NP. It is apparent that *Acan* expression in the IAF and the NP is predominantly regulated by SOX9 and SOX5/6. In contrast, predominant PAX1/9 and SCX expression in the OAF and IVD defects of mice lacking these genes suggest the importance of these weak transactivators in transcriptional regulation of *Acan* in the OAF. Since the *UE* accounts for ~40% of endogenous *Acan* expression, it is reasonable to speculate that PAX1/9 regulates *Acan* expression directly through other *Acan* enhancers or indirectly by affecting the expression of downstream genes that regulate *Acan* expression. So far, we have revealed that the SOX9-driven transactivation in the *Nkx3*.*2* promoter is modulated by PAX1/9 through competition for overlapping binding sites and physical interactions. Genome wide identification of *Acan* enhancers and ChIP Seq studies will reveal whether a similar mechanism is found in other enhancers to regulate *Acan* transactivation.

## Methods

### Animals

C57BL/6 mice and Wistar rats were purchased from Shimizu Laboratory Supplies (Kyoto, Japan). Fertile White Leghorn chicken eggs were obtained from Takeuchi Farm (Nara, Japan). All animal experimental procedures used in this study were approved by the Animal Care Committee of the Institute for Frontier Life and Medical Sciences, Kyoto University and conformed to institutional guidelines for the study of vertebrates.

### Generation of transgenic mice

To generate the *Col2a1-Pax1* construct, mouse *Pax1* cDNA was amplified by PCR using the primers listed in Supplementary Table 2 and inserted into the NotI site of the *pCol2a1* vector containing the promoter and intronic enhancer of the rat *Col2a1* gene^28,29^. The purified *Col2a1-Pax1* transgene was injected into the pronuclei of fertilized eggs from C57BL/6× C3H F1 mice. Injected founder embryos were dissected and analyzed from E12.5 to E16.5. Transgene positive embryos were identified by PCR using the primers listed in Supplementary Table 2 and genomic DNA extracted from the yolk sac.

### Generation of mouse lines by CRISPR/Cas9-mediated gene editing

For CRISPR/Cas9-mediated gene editing, crisprRNAs (crRNAs) were designed using the CRISPRdirect website^30^ and chemically synthesized and purified by Fasmac (Kanagawa, Japan). The crRNAs, trans-activating crRNA, and Cas9 protein were microinjected into the cytoplasm or pronuclei of fertilized eggs obtained from C57BL/6 mice. Injected eggs were transferred into the oviducts of pseudopregnant surrogate ICR female mice. Deletions in genomic DNA were identified by direct sequencing of the PCR products amplified using the primers listed in Supplementary Table 2. Mouse lines were established from founder mice carrying deletions in targeted DNA sites.

### Tissue sections

For the preparation of frozen sections of embryonic tissues, mouse embryos were fixed in 4% paraformaldehyde dissolved in PBS (PFA/PBS) for 3 h, infiltrated with 18% sucrose/PBS, embedded in Tissue-Tek O.C.T. compound (Sakura Finetek Japan), cut at a thickness of 8 μm, and mounted onto glass slides.

### Histological staining

For alcian blue or toluidine blue staining of frozen sections, the sections were fixed with 4% PFA/PBS for 5 min and stained with 1% alcian blue 8GX (Sigma, St. Louis, MO, USA) at pH 1 or 0.05% toluidine blue O (Sigma) at pH 4 for 5 min. For whole mount alcian blue staining, embryos were fixed in 5% trichloroacetic acid for 3 h and washed with distilled water. The embryos were then stained with 0.1% alcian blue 8GX (Sigma) in 70% ethanol containing 0.1% HCl for 3 h and destained in 70% ethanol containing 0.1% HCl. After dehydrating twice in 100% ethanol for 1 h, the samples were cleared and stored in methyl salicylate.

### Immunostaining

Sections for the detection of PAX1, PAX9, SOX5, and SOX6 were boiled in 10 mM sodium citrate at pH 6 for 10 min. Sections for the detection of ACAN were pretreated with 0.2 U/mL chondroitinase ABC (Sigma) at 37 °C for 30 min. After blocking with 3.2% skim milk/PBS, the sections were incubated with primary antibodies for 16 h, washed, and then incubated with appropriate secondary antibodies conjugated with Alexa Fluor 488 or 594 purchased from Thermo Fisher Scientific (Waltham, MA, USA). Nuclei were counterstained with 4′,6-diamidino-2-phenylindole (DAPI; Sigma). The primary antibodies used were anti-ACAN (Merck Millipore, Burlington, MA, USA, AB1031; 1:500), anti-PAX1 (Santa Cruz Biotechnology, Santa Cruz, CA, USA, sc-7744; 1:500), anti-PAX9 (Active Motif, Carlsbad, CA, USA, 61077; 1:500), anti-SOX5 (Abcam, Cambridge, UK, ab94396; 1:500), anti-SOX6 (Abcam, ab30455; 1:500), and anti-SOX9 (Merck Millipore, AB5535; 1:800). The images were captured under a Leica DMRXA microscope equipped with a Leica DC500 camera (Leica Microsystems).

### *In situ* hybridization

For RNA probes, the complementary DNAs (cDNAs) for *Acan*, *type II collagen* (*Col2a1*), and *Pax1* were amplified by reverse transcription-polymerase chain reaction (RT-PCR) based on sequence information available in GenBank (*Acan*, L07049; *Col2a1*, NM031163; *Pax1*, NM008780). Mouse *Sox9* and *Col10a1* cDNAs were generously given by Dr. Gerd Scherer (University of Freiburg)^28^ and Dr. Bjorn R Olsen (Harvard Medical School)^31^, respectively. The RNA probes were transcribed from the linearized plasmids with a digoxigenin (DIG) RNA labelling kit (Roche, Mannheim, Germany). The frozen sections were fixed in 4% PFA/PBS for 10 min, washed with PBS, and treated with 10 μg/mL proteinase K (Thermo Fisher Scientific) for 15 min. After post-fixation with 4% PFA/PBS for 10 min, the sections were carbethoxylated twice in 0.1% DEPC/PBS and hybridized with DIG-labelled RNA probes diluted in 50% formaldehyde/5 × SSC containing 40 μg/mL salmon sperm DNA at 55 °C for 16 h. To detect DIG-labelled RNA probes, immunological detection was performed with anti-DIG-AP Fab fragment (Roche) and BM purple (Roche).

### Vector construction

The entire coding regions of mouse *Pax1*, mouse *Pax9*, human *Sox5*, human *Sox6*, and mouse *Sox9* were obtained from *RCAS(A)-Pax1*, *RCAS(A)-Pax9*^10^, *pMXs-gw/hSOX5*, *pMXs-gw/hSOX6*^32^, and *pCMV-SPORT6-Sox9* (OriGene Technologies, Rockville, USA), respectively. To construct expression plasmids, each DNA fragment was inserted into *pcDNA3* or *CAG-IRES-EGFP* vectors. To generate N-terminal FLAG-tagged PAX1 and PAX9, each DNA fragment was amplified by PCR to add *Not*I sites and inserted into the *pcDNA3-FLAG* vector. A 359-bp *UE* was amplified by PCR from mouse genomic DNA with forward and reverse primers designed with *BamH*I and *Bgl*II sites, respectively, and then PCR products were cloned into the *pCR4-TOPO* vector (Thermo Fisher Scientific). Four tandem copies of *UE* were obtained by ligation of monomers digested with *BamH*I and *Bgl*II. To construct *pGL4*.*23[luc2/minP]-1xUE-Luc* or *pGL4*.*23[luc2/minP]-4xUE-Luc*, one copy or four tandem copies of *UE* were inserted into the *pGL4*.*23[luc2/minP]* vector (Promega, Madison, WI, USA) containing a minimal promoter and the firefly luciferase reporter gene. The 508-bp *I12E* or 1109-bp *Nkx3*.*2-P* were amplified by PCR from mouse genomic DNA and inserted into the *pGL4*.*23[luc2/minP]* or *pGL3-Basic* vector (Promega) containing the firefly luciferase reporter gene, respectively. The PCR primers for constructs are listed in Supplementary Table 2.

### Cell culture and transient transfection

AF cells were isolated from the tail IVDs of 4-week-old male Wistar rats. AFs were minced and partially digested with 0.2% collagenase (Roche) and 0.1% trypsin (BD Biosciences) at 37 °C for 20 min. The tissue fragments were placed onto 10-cm cell culture dishes (BD Biosciences) coated with type I collagen (KOKEN, Tokyo, Japan), and the cultures were maintained in MF-start (TOYOBO, Osaka, Japan). At 80% confluence, the cells grown from AFs were passaged twice and grown in Dulbecco’s modified Eagle’s medium (DMEM; Sigma) containing 10% fetal bovine serum (FBS). Chondrocytes were isolated from the distal tibiotarsal cartilage of chicken embryos at stage 41, as previously described^33^. Human embryonic kidney (HEK) 293T cells and NIH3T3 cells were cultured in DMEM containing 10% FBS. Transfection of plasmids into the cells was performed using Lipofectamine LTX Reagent (Thermo Fisher Scientific) or Lipofectamine 3000 Reagent (Thermo Fisher Scientific). Cell cultures were maintained in a humidified atmosphere of 5% CO_2_ in air.

### Gene silencing experiments

Small interfering RNA (siRNA) oligonucleotide duplexes were purchased from GE Healthcare Life Sciences (Little Chalfont, UK). *Pax1* was knocked down using *siPax1-a* (J-092982-12) and *siPax1-b* (J-092982-17), included in the ON-TARGET plus Rat Pax1 siRNA-Set of four (GE Healthcare, LQ-092982-02-002). For the control experiment, siGENOME non-targeting siRNA Pool number 1 (GE Healthcare, D-001206-13-05) was used. Transfection of siRNA into AF cells was performed with Lipofectamine 3000 Reagent (Thermo Fisher Scientific) according to the manufacturer’s instructions. For lentivirus-mediated knockdown of *Pax1*, lentiviral particles were produced using HIV-based lentiviral vector constructs purchased from SBI (Palo Alto, CA, USA). The short hairpin RNA (shRNA) sequences for *shPax1-a* and *shPax1-b* were created based on the sequences of J-092982-11 and J-092982-17 included in the ON-TARGET plus Rat Pax1 siRNA-Set of four (GE Healthcare, LQ-092982-02-002), respectively. For the control experiment, the non-targeting sequence (UAAGGCUAUGAAGAGAUAC) included in siGENOME non-targeting siRNA Pool no. 1 (GE Healthcare, D-001206-13-05) was used. The target sequences were connected by a loop sequence (CUUCCUGUCAGA) and restriction enzyme sites and then inserted into the lentivirus-mediated shRNA-expressing vector *pGreenPuro* (SBI, SI505A-1). The lentiviral particles were produced in Lenti-X 293T cells (TaKaRa Bio, Shiga, Japan) and concentrated with PEG-it Virus Precipitation Solution (SBI), according to the manufacturer’s instructions.

### Chondrogenic induction

For induction of chondrogenic differentiation, aliquots of 1.5 × 10^5^ AF cells were pelleted in 15-mL conical tubes and maintained for 21 days in 0.5 mL DMEM containing 6.25 μg/mL insulin, 6.25 μg/mL transferrin, 6.25 ng/mL sodium selenite, 1.25 mg/mL bovine serum albumin, 50 μg/mL l-ascorbic acid, 1 μM dexamethasone, and 10 ng/mL recombinant human TGF-β1, as previously described^34^.

### Immunoprecipitation and Immunoblotting

Nuclear extracts prepared from HEK293T cells transfected with a total of 10 μg of plasmids were incubated with 5 μg of anti-FLAG M2 antibody (Sigma, F3165) in the immunoprecipitation buffer [0.1% NP-40, 1 mM EDTA, 100 mM NaCl, 50 mM Tris-HCl (pH 7.5)] at 4 °C for 4 h with gentle rocking. Twenty-five microliters of Protein G magnetic beads (Active motif, 53033) was then added to the mixture and incubated at 4 °C for another 1 h with gentle rocking. After incubation, the beads were washed twice with the immunoprecipitation buffer and then with ice-cold PBS twice. The bound protein was eluted with 20 μL of 2 × SDS sample buffer [100 mM Tris-HCl (pH 6.8), 4% SDS, 100 mM DTT, 20% glycerol] and boiled for 5 min. The eluate and nuclear extracts were separated by SDS-PAGE and transferred onto Immobilon-P transfer membrane (Merk Millipore). The membranes were preincubated with 3.2% skim milk in Tris-buffered saline (TBS) for 30 min and incubated with anti-SOX9 antibody (Merck Millipore, AB5535; 1:2000) at 4 °C overnight. Blots were washed with TBS containing 0.05% Tween 20 and incubated with Trueblot HRP-conjugated anti-rabbit IgG (Rockland, 18-8816-31). Antibodies were detected using ImmunoStar Zeta (Wako, 291-72401) and ImageQuant LAS 4000mini (GE healthcare).

### RT-PCR and quantitative RT-PCR (qRT-PCR) analysis

For RNA extraction from the costal cartilage and the AF, tissues dissected from the rib cage and the tail, respectively, were immersed in RNA*later* solution (Thermo Fisher Scientific). Total RNA was extracted from AF tissues or AF cells using RNeasy Plus Mini Kit (Qiagen, Hilden, Germany), according to the manufacturer’s instructions. The costal cartilage was frozen in liquid nitrogen and crushed using SK-mill (Tokken, Chiba, Japan), and then the total RNA was purified by RNeasy MinElute Cleanup kit (Qiagen) after phenol-chloroform extraction. One or two hundred nanograms of total RNA was used to synthesize cDNA with a PrimeScript RT reagent Kit (Takara Bio). RT-PCR was performed with Takara Ex Taq (Takara Bio) and the same primers for qRT-PCR listed in Supplementary Table 3. qRT-PCR was performed using SYBR Premix Ex Taq II (Takara Bio) on a StepOne instrument (Thermo Fisher Scientific). Relative mRNA expression was normalized to that of *18 S* rRNA and calculated using the 2^−ΔΔCT^ method.

### Chromatin immunoprecipitation (ChIP) assay

ChIP assays were performed using a ChIP-IT Express Magnetic Chromatin Immunoprecipitation kit (Active Motif) following the manufacturer’s protocol. Sheared chromatin prepared from AF cells was immunoprecipitated using anti-PAX1 (Santa Cruz Biotechnology, sc-7744), anti-SOX9 (Millipore, AB5535), or normal goat IgG (Santa Cruz Biotechnology, sc-2028) antibodies. The sequences of *Acan-UE*, *Acan-I12E*, *Nkx3*.*2-P*^10^, and *Col2a1-E*^5^ were detected by PCR using Takara Ex Taq (Takara Bio) and the ChIP primers listed in Supplementary Table 2. The amplification products were electrophoresed on a 2% agarose gel and visualized by ethidium bromide.

### Luciferase reporter assay

HEK293T or NIH3T3 cells seeded in 96-well microtiter plates were transfected with 50 ng of the *pGL4*.*23* construct, 50 ng of *pcDNA3* expression vectors, and 1 ng of *pGL4*.*74*[*hRluc/TK*] vector (Promega) containing the HSV-TK promoter and *Renilla* luciferase. Chondrocytes seeded in 96-well microtiter plates were transfected with 100 ng of the *pGL4*.*23* construct, 100 ng of *pcDNA3* expression vectors, and 1 ng of *pGL4*.*74*[*hRluc/TK*] vector. After 24 h, luciferase activities were measured using the Pica gene Dual luciferase kit (Toyo ink, Tokyo, Japan) and a microplate luminometer (Centro XS^3^ LB960, Berthold Technologies, Bad Wildbad, Germany). Reporter construct activity was normalized by comparison with activity from the *Renilla* luciferase construct. All experiments were performed in triplicate.

### Gel shift assay

Protein-DNA binding was assayed with the Gelshift Chemiluminescent EMSA kit (Active Motif) following the manufacturer’s protocol. For the SOX9 protein, the binding reactions were carried out in 20 μL of binding buffer containing 1 μg of poly(dG-dC)·poly(dG-dC) and 20 μg of BSA. To generate the DNA probes or competitors above 100 bp in size, double-stranded DNA (dsDNA) fragments were amplified by PCR with the biotin-labelled or unlabeled PCR primers listed in Supplementary Table 2, respectively. The biotin-labelled DNA probes less than 100 bp in size were prepared from synthesized DNA oligonucleotides using the Biotin 3′ End DNA Labeling kit (Thermo Fisher Scientific). Nuclear proteins were isolated using the Nuclear Extract Kit (Active Motif) from HEK293T cells transfected with *pcDNA3* vectors to express FLAG-PAX1, FLAG-PAX9, and SOX9 protein or *pCAG-IRES-EGFP* vectors to express PAX1 and SOX9 protein. Five micrograms of anti-FLAG M2 monoclonal antibody (Sigma, F3165) was used for each reaction of the antibody with the nuclear protein.

### Statistical analysis

*P*-values were calculated by *t*-test or one-way analysis of variance using the SPSS software package (SPSS 21.0). The data were considered statistically significant when the *P* value was < 0.05.

## Supplementary information

Supplementary Information
