# Supplementary material for: Differential transactivation of the upstream aggrecan enhancer regulated by PAX1/9 depends on SOX9-driven transactivation
Source: Sci Rep. 2019 Mar 14;9:4605. doi: 10.1038/s41598-019-40810-4 (PMC6418084; doi:10.1038/s41598-019-40810-4)
Supplement: Supplementary file 1 — Supplementary Information [file 41598_2019_40810_MOESM1_ESM.docx]

**Differential transactivation of the upstream aggrecan enhancer regulated by PAX1/9 depends on SOX9-driven transactivation**

Aki Takimoto**^1^**, Chikara Kokubu**^2^**, Hitomi Watanabe**^3^**, Tetsushi Sakuma**^4^**, Takashi Yamamoto**^4^**, Gen Kondoh**^3^**, Yuji Hiraki**^1^**, and Chisa Shukunami^5^*

^1^Laboratory of Cellular Differentiation, Institute for Frontier Life and Medical Sciences, Kyoto University, Kyoto 606-8507, Japan

^2^Department of Genome Biology, Graduate School of Medicine, Osaka University, Suita, Osaka, Japan

^3^ Laboratory of Integrative Biological Science, Institute for Frontier Life and Medical Sciences, Kyoto University, Kyoto 606-8507, Japan

^4^ Department of Mathematical and Life Sciences, Graduate School of Science, Hiroshima University, Higashi-Hiroshima, Hiroshima 739-8526, Japan

^5^ Department of Molecular Biology and Biochemistry, Division of Dental Sciences, Graduate School of Biomedical and Health Sciences, Hiroshima University, Hiroshima 734-8553, Japan

*Address all correspondence to Chisa Shukunami, DDS, Ph.D.

Department of Molecular Biology and Biochemistry, Division of Dental Sciences, Graduate School of Biomedical & Health Sciences, Hiroshima University, Hiroshima 734-8553, Japan

# E-mail: [shukunam@hiroshima-u.ac.jp](mailto:shukunam@hiroshima-u.ac.jp)

# Methods for Supplementary Fig. 3

**Skeletal preparation**

For double staining with alizarin red and alcian blue, mouse embryos were dehydrated with ethanol and stained with 0.015% alcian blue 8 GX (sigma) and cleared with 2% potassium hydroxide. The embryos were then stained with 0.05% alizarin red S (Wako) in 1% KOH and cleared with 1% KOH.

**Supplementary Fig. 1. Expression levels of *Pax1*, *Sox5*, *Sox6*, and *Sox9* in the AF cells cultured under non-inductive or chondro-inductive conditions.**

Total RNA was extracted from the AF cells cultured under non-inductive or chondro-inductive conditions, shown in Fig. 2. The expression levels of *Pax1*, *Sox5*, *Sox6*, or *Sox9* were examined by qRT-PCR analysis. Relative expression levels were normalized to that in the non-inductive condition and reported as mean ± s.d. qRT-PCR data represent the average of three independent experiments. ****P* < 0.001 versus control.

**Supplementary Fig. 2. Expression level of *Pax9* in the pellets of AF cells.**

Total RNA was extracted from pellets infected with control, *shPax1-a*, or *shPax1-b* lentivirus. Relative expression level of *Pax9* was examined by qRT-PCR analysis, was normalized to that in the control, and reported as mean ± s.d. qRT-PCR data represent the average of three independent experiments. **P* < 0.05 versus control.

**Supplementary Fig. 3.** **Lack of endochondral bone formation in *Col2a1-Pax1* transgenic mouse embryos.**

(a-d) Cartilage models are visualized by whole mount alcian blue staining of wild type (*Wt*) (a,b) and *Col2a1-Pax1* transgenic (*Tg*) (c,d) mouse embryos at E14.5. Lateral (a,c) and dorsal (b,d) views are shown. Arrows in (c) indicate the curvatures in the vertebral column of *Tg* embryo. (e-h) *Wt* and *Tg* embryos at E16.5 shown in (e) and (g) were processed for whole mount alcian blue and alizarin red staining (f,h). Lateral views of *Wt* (e,f) and *Tg* (g, h) embryos are shown.

**Supplementary Fig. 4. PAX1/9-binding site in intron 12 of the mouse *Acan* gene.**

(a) Genomic structure of mouse *Acan*, consisting of 17 exons (black boxes). Black arrowheads indicate the positions of *UE,* located approximately 10 kb upstream of the transcription start site, and of *I12E,* located in intron 12. (b) A 508-bp *I12E* sequence containing the predicted PAX2-binding site (underlined). Bold letters indicate the oligonucleotide sequence used for the gel shift assays shown in (c-e). (c) Gel shift assay was performed using 20 fmol of a biotin-labelled dsDNA probe, 20 pmol of an unlabeled dsDNA fragment, and nuclear extracts (N.E.) of HEK293T cells transfected with *pcDNA3* empty vector (N.E. Control) or *pcDNA3-FLAG-Pax1* (N.E. FLAG-PAX1). (d,e) Gel shift assay was performed using 100 fmol of a biotin-labeled dsDNA probe and nuclear extracts of HEK293T cells transfected with *pcDNA3* empty vector (N.E. Control), *pcDNA3-FLAG-Pax9* (N.E. FLAG-PAX9), or *pcDNA3-Sox9* (N.E. SOX9). A shifted band represents the interaction between the biotin-labelled 240-269 of *I12E* and FLAG-PAX1 or FLAG-PAX9*.* A supershifted band was generated by the addition of anti-FLAG antibody (Anti-FLAG Ab.). A shifted and a supershifted band are shown with an arrow. Non-specific bindings are shown with an open arrowhead. s, shifted band; ss, supershifted band.

**Supplementary Fig. 5. Transactivation of the *UE* in chondrocytes.**

Dual luciferase assays were performed with chondrocytes. Cells were co-transfected with reporter plasmids (*pGL4.74 [hRluc/TK]* and *pGL4.23[luc2/minP]* vectors) and *pcDNA3* empty vector. The *pGL4.23[luc2/minP]* vectors used for the assays were *pGL4.23[luc2/minP]* empty vector (*pGL4.23-Luc*)*, pGL4.23[luc2/minP]-1xUE-Luc* (*1xUE-Luc*), or *pGL4.23[luc2/minP]-4xUE-Luc* (*4xUE-Luc*). The firefly and *Renilla* luciferase activities were measured 24 h after transfection. Values were normalized using a *pGL4.74[hRluc/TK]* vector and are presented as fold induction relative to *pGL4.23-Luc*. Graphs show a representative experiment out of at least three. Each bar represents the average of three independent transfections (means ± s.d.). ****P* < 0.001 versus *pGL4.23-Luc*.

**Supplementary Fig. 6. Identification of the PAX1-binding site in the *UE*.**

(a,b) Gel shift assays were performed using 100 fmol of biotin-labelled oligonucleotides shown in Supplementary Table 1 and nuclear extracts (N.E.) of HEK293T cells transfected with *pcDNA3* empty vector (N.E. Control), *pcDNA3-FLAG-Pax1* (N.E. FLAG-PAX1) (a), or *pcDNA3-FLAG-Pax9* (N.E. FLAG-PAX9) (b). The specific bindings of the protein to the oligonucleotides were confirmed by antibody supershifts with anti-FLAG antibody (Antibody). A shifted and a supershifted band are shown with an arrow. Non-specific bindings are shown with open arrowheads. The specific binding between FLAG-PAX1 and the oligonucleotide was detectable in *wt*, *m1*, *m2*, *m3*, *m4*, or *m9*. The specific binding was also detectable between FLAG-PAX9 and *wt* or *m3*, but undetectable between FLAG-PAX9 and *m7*. s, shifted band; ss, supershifted band.

**Supplementary Fig. 7 Full images of gel electrophoresis.**

Full images of gel electrophoresis are shown. The cropped images are enclosed with dotted lines.

**Supplementary Table 1. *Wild type* (*wt*) and mutated (*m1*-*m9*) nucleotide sequences from 301 to 355 of the *UE*.**

|  | | Sequence (5’ - 3’) |
| --- | --- | --- |
| *wt* | TTTCAAACCTGGCAGGGTGAATAGGTGCCTTGTGTTCAGGCTTATGACTCTGGAA | |
| *m1* | TTTCAAACCTGGCAAAAAAAATAGGTGCCTTGTGTTCAGGCTTATGACTCTGGAA | |
| *m2* | TTTCAAACCTGGCAGGGTGAAAAAAAGCCTTGTGTTCAGGCTTATGACTCTGGAA | |
| *m3* | TTTCAAACCTGGCAGGGTGAATAGGTGAAAAATGTTCAGGCTTATGACTCTGGAA | |
| *m4* | TTTCAAACCTGGCAGGGTGAATAGGTGCAAAAAGTTCAGGCTTATGACTCTGGAA | |
| *m5* | TTTCAAACCTGGCAGGGTGAATAGGTGCCAAAAATTCAGGCTTATGACTCTGGAA | |
| *m6* | TTTCAAACCTGGCAGGGTGAATAGGTGCCTTGTGTTCAAAAATATGACTCTGGAA | |
| *m7* | TTTCAAACCTGGCAGGGTGAATAGGTGCCTTGTGTTCAGGCTTAAAAATCTGGAA | |
| *m8* | TTTCAAACCTGGCAGGGTGAATAGGTGCCTTGTGTTCAGGCTTATGACTCAAATT | |
| *m9* | TTTCAAACCTGGCAGGGTGAATAGGTGCCTTGTGTTCAGGCTTATGACTCTAATT | |

Underlined and boxed regions in the *wt* sequence are the SOX9-binding sites and the PAX1-binding site, respectively, as revealed by gel shift assays in Supplementary Fig. 6. The mutant nucleotides in *m1*-*m9* are shown in red.

**Supplementary Table 2. Primers for constructs, DNA fragments for gel shift assays, ChIP PCR, and genotyping.**

|  | |  | Sequence (5’ - 3’) | |
| --- | --- | --- | --- | --- |
| *Col2a1-Pax1* | | F  R | ataagaatgcggccgcgcgcgatggagcagacg  ataagaatgcggccgcgcccctaggaggtcgagg | |
| *pcDNA3-*  *FLAG-Pax1* | | F  R | ATAAGAATGCGGCCGCGCGATGGAGCAGACG  ATAAGAATGCGGCCGCCCCTAGGAGGTCGAGG | |
| *pcDNA3-*  *FLAG-Pax9* | | F | ATAAGAATGCGGCCGCGATGGAGCCAGCCTTCGGGGAGG | |
|  |  | R | ATAAGAATGCGGCCGCTCAGAGTGCAGAAGCGGTCACAG | |
| *pGL4.23-* | | F | GGATCCGTCCAGAATGGAAGGGACCAAG | |
| *UE-Luc* | | R | AGATCTGCTTTTCCAGAGTCATAAGCCTG | |
| *pGL4.23-* | | F | CTGCTAGAATGACACGGCATC | |
| *I12E-Luc* | | R | AAAGCACCACAAGTGGAGTGC | |
| *pGL3-Basic-* | | F | CTAGCTAGCCCCAAGGAGACGAATACCCAG | |
| *Nkx3.2-P-Luc* | | R | CCCAAGCTTGATCCGCGCCGACCCTCACCC | |
| DNA fragments of *UE* | 1-130 | F | GTCCAGAATGGAAGGGACC | |
|  |  | R | TGCGCTGTTTATGTGGGTG | |
|  | 118-259 | F | CATAAACAGCGCATCCACAAA | |
|  |  | R | CACTGTGCATCTTTTCAAATT | |
|  | 249-359 | F | GATGCACAGTGTACTTGGG | |
|  |  | R | GCTTTTCCAGAGTCATAAG | |
| ChIP PCR | *Acan-*  *UE* | F | TGTCTGAAATTTGAAAAGAT |  |
|  |  | R | TCGTAAGCCTGAACACAAGG |  |
|  | *Acan-*  *I12E* | F | ATGTCCTTTCCATGACCCTAG |  |
|  |  | R | GGAGCAAGAAAGAAAGCATGGACC |  |
|  | *Nkx3.2-P* | F | TCAACCTAGCTTTTTAAGTC |  |
|  |  | R | AAGTTTCTCAGCTGTTTCTC |  |
|  | *Col2a1-E* | F | AGGCTTGTTTGCGTTGAGGGATTG |  |
|  |  | R | GCCGTGCGGCATAAGTGATTCTTT |  |

Supplementary Table 2. Continued

|  |  |  | Sequence (5’ - 3’) |
| --- | --- | --- | --- |
| Genotyping | *Col2a1-Pax1 Tg* mouse | F  R | gcgcgatggagcagacg  gcccctaggaggtcgagg |
|  | *UE-deletion* mouse | F  R | AATTTCCAATGCGAGAAACTG  TCACCGATTGGAAGGAGATG |

**Supplementary Table 3. Primers for RT-PCR and qRT-PCR.**

| Species |  |  | Sequence (5’ - 3’) |
| --- | --- | --- | --- |
| rat | *Acan* | F | CATCTGAGCAGCATCGTCACC |
|  |  | R | GTTCTGTCATTCAGACCGATCCAC |
|  | *Col1a2* | F | ACTCAGCCACCCAGAGTGGAA |
|  |  | R | TTGACAGGTTGGGCCTGGA |
|  | *Col2a1* | F | GAGGGCAACAGCAGGTTCAC |
|  |  | R | GCCCTATGTCCACACCAAATTC |
|  | *Nkx3.2* | F | TTGAATTGAACCTCGAAGCG |
|  |  | R | GCAGGTTTCTGAAAGGGGCT |
|  | *Ocn* | F | GGTGCAGACCTAGCAGACACCA |
|  |  | R | AGGTAGCGCCGGAGTCTATTCA |
|  | *Pax1* | F | CACATTCAGTCAGCAACATTCTGG |
|  |  | R | AAGGCGGCACGATTCACAC |
|  | *Pax9* | F | CATTCGGCTTCGCATCGTG |
|  |  | R | CTCCCGGCAAAATCGAACC |
|  | *Sox5* | F | cctctgtcccagcagcgtta |
|  |  | R | GGTGACAGCATCGTGGTCATTTA |
|  | *Sox6* | F | AGCTGATGCGCTCACGAAG |
|  |  | R | GATGGTGTGGTCGTTGCCATA |
|  | *Sox9* | F | AGGAAGCTGGCAGACCAGTA |
|  |  | R | ACGAAGGGTCTCTTCTCGCT |
|  | *18S rRNA* | F | AAGTTTCAGCACATCCTGCGAGTA |
|  |  | R | TTGGTGAGGTCAATGTCTGCTTTC |
| mouse | *Acan* | F | AGTGGATCGGTCTGAATGACAGG |
|  |  | R | AGAAGTTGTCAGGCTGGTTTGGA |
|  | *Sox9* | F | ACGGCTCCAGCAAGAACAAG |
|  |  | R | TTGTGCAGATGCGGGTACTG |
|  | *18S rRNA* | F | TTCTGGCCAACGGTCTAGACAAC |
|  |  | R | CCAGTGGTCTTGGTGTGCTGA |
